# Imaging Neuroinflammation – from Bench to Bedside

**DOI:** 10.4172/2155-9899.1000226

**Published:** 2014-06-30

**Authors:** Benjamin Pulli, John W Chen

**Affiliations:** Center for Systems Biology and Department of Radiology, Massachusetts General Hospital and Harvard Medical School, 185 Cambridge Street, Boston, MA 02114, USA

**Keywords:** Molecular imaging, Neuroinflammation, MRI, PET, Inflammation, CNS, Brain

## Abstract

Neuroinflammation plays a central role in a variety of neurological diseases, including stroke, multiple sclerosis, Alzheimer’s disease, and malignant CNS neoplasms, among many other. Different cell types and molecular mediators participate in a cascade of events in the brain that is ultimately aimed at control, regeneration and repair, but leads to damage of brain tissue under pathological conditions. Non-invasive molecular imaging of key players in the inflammation cascade holds promise for identification and quantification of the disease process before it is too late for effective therapeutic intervention. In this review, we focus on molecular imaging techniques that target inflammatory cells and molecules that are of interest in neuroinflammation, especially those with high translational potential. Over the past decade, a plethora of molecular imaging agents have been developed and tested in animal models of (neuro)inflammation, and a few have been translated from bench to bedside. The most promising imaging techniques to visualize neuroinflammation include MRI, positron emission tomography (PET), single photon emission computed tomography (SPECT), and optical imaging methods. These techniques enable us to image adhesion molecules to visualize endothelial cell activation, assess leukocyte functions such as oxidative stress, granule release, and phagocytosis, and label a variety of inflammatory cells for cell tracking experiments. In addition, several cell types and their activation can be specifically targeted *in vivo*, and consequences of neuroinflammation such as neuronal death and demyelination can be quantified. As we continue to make progress in utilizing molecular imaging technology to study and understand neuroinflammation, increasing efforts and investment should be made to bring more of these novel imaging agents from the “bench to bedside.”

## Introduction

Neuroinflammation plays a central role in a variety of neurological diseases, including cerebrovascular disease (e.g., stroke), demyelinating diseases (e.g., multiple sclerosis, MS), neurodegeneration (e.g., Alzheimer’s disease), and malignant CNS neoplasms (e.g., glioblastoma multiforme), among many others [1–6]. Depending on the specific condition, different cell types and molecular mediators (e.g., cytokines, chemokines) participate in a cascade of events in the brain that is ultimately aimed at control, regeneration and repair, but leads to damage of brain tissue under pathological conditions [2,7,8]. Activation of the resident leukocyte in the brain, the microglia, presents one of the hallmarks of neuroinflammation, which is often accompanied by blood-brain barrier (BBB) breakdown, cytokine and chemokine release, as well as blood-borne leukocyte infiltration. Damage to neurons and myelin sheaths can be caused by myeloid cells through oxidative stress, phagocytosis, and proteases, and by lymphocytes through antibody-dependent cell-mediated cytotoxicity or cytolytic granule release [9].

Therefore, neuroinflammation is a highly relevant diagnostic and therapeutic target, but several characteristics of the brain make both goals more difficult than at other sites in the body. First, the brain has the BBB, which prevents most molecules from entering the brain. Thus, imaging probe and drug design needs to bypass this challenge. Second, cranial bones complicate direct access to the brain for diagnostic (e.g., biopsies for tissue sampling) or therapeutic (e.g., surgery) interventions, and even distorts signal of certain (e.g., optical) imaging techniques. Lastly, the brain has very limited regeneration capacity, which makes secondary and tertiary prevention more difficult, so early diagnosis is of utmost importance.

Although neuroinflammatory diseases have been visualized with a variety of imaging techniques and agents, the majority of imaging in current clinical practice is done with methods not specifically targeted at molecular mediators of the immune system. For example, gadolinium-enhanced magnetic resonance (MR) imaging detects BBB breakdown but is not specifically targeted at the molecules and cells that facilitate this process. It is therefore not surprising that BBB breakdown on MR imaging and inflammation do not always correlate [10].

**Figure 1:**
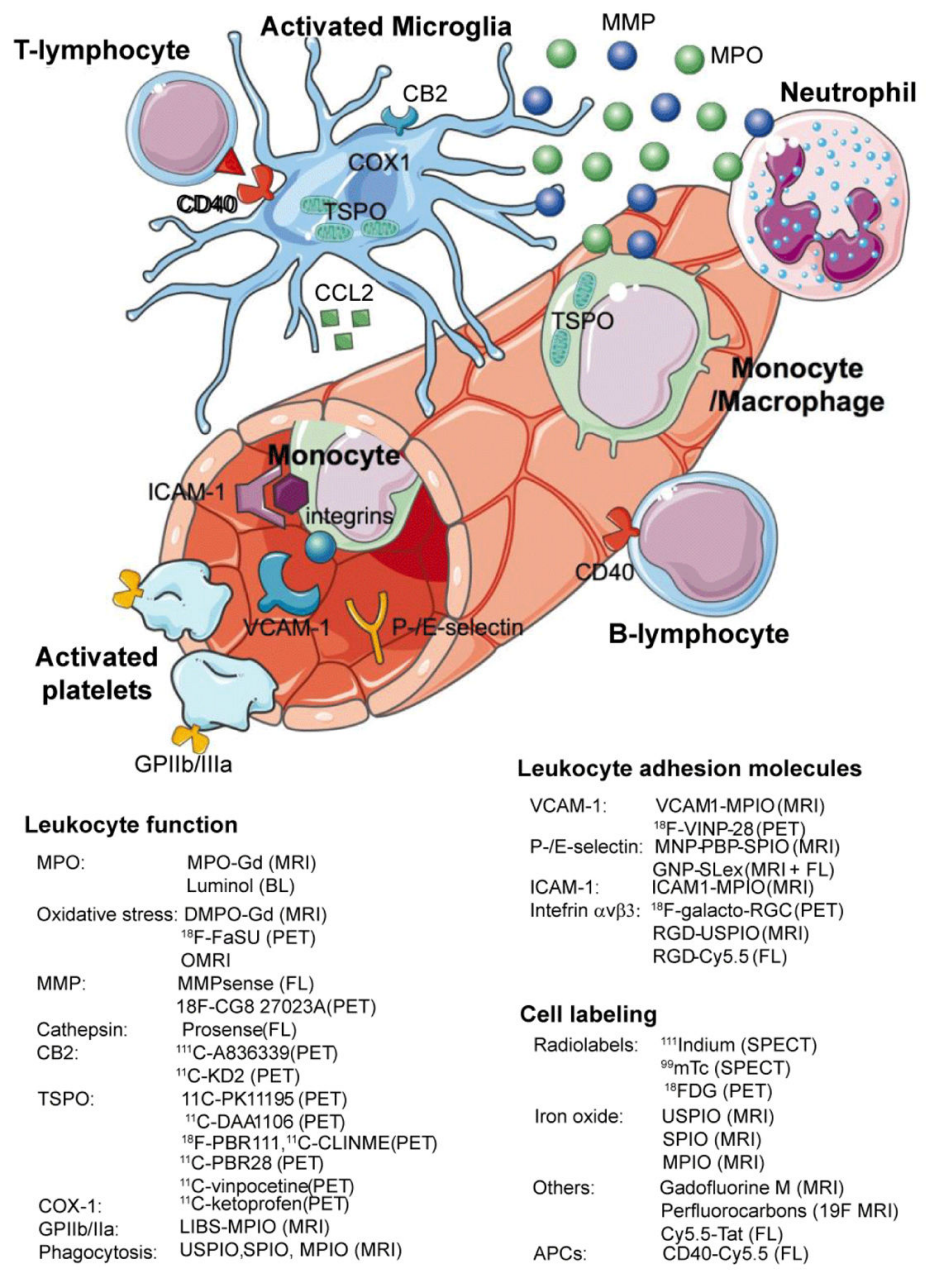
Targets and probes in molecular imaging of neuroinflammation. Key cellular players in neuroinflammation are activated microglia, monocytes/macrophages, neutrophils, T-lymphocytes and B-lymphocytes. Microglia are resident brain leukocytes, and upregulate translocator protein (TSPO), cyclooxygenase 1 (COX1), and cannabinoid receptor 2 (CB2) under inflammatory conditions. Blood-borne leukocytes extravasate into the brain through interaction of cell surface integrins with specific endothelial adhesion molecules (e.g., ICAM-1, VCAM-1, P-/E-selectin). Once in the subendothelial space, exposure to chemokines (e.g. CCL2 released by microglia) further directs them towards their target. Stimulated cells then secrete effector molecules (e.g., matrix metalloproteinases [MMPs] and myeloperoxidase [MPO]), which trigger axonal damage and/or demyelination. Cell interaction between antigen-presenting cells (APCs, e.g., B-lymphocytes, microglia, dendritic cells) is mediated via CD40 amongst other molecules. Activated platelets can also produce reactive oxidative species and trigger thrombosis. BL: Bioluminescence imaging; FL: Fluorescence Imaging (Adapted from Servier Medical Art).

**Figure 2:**
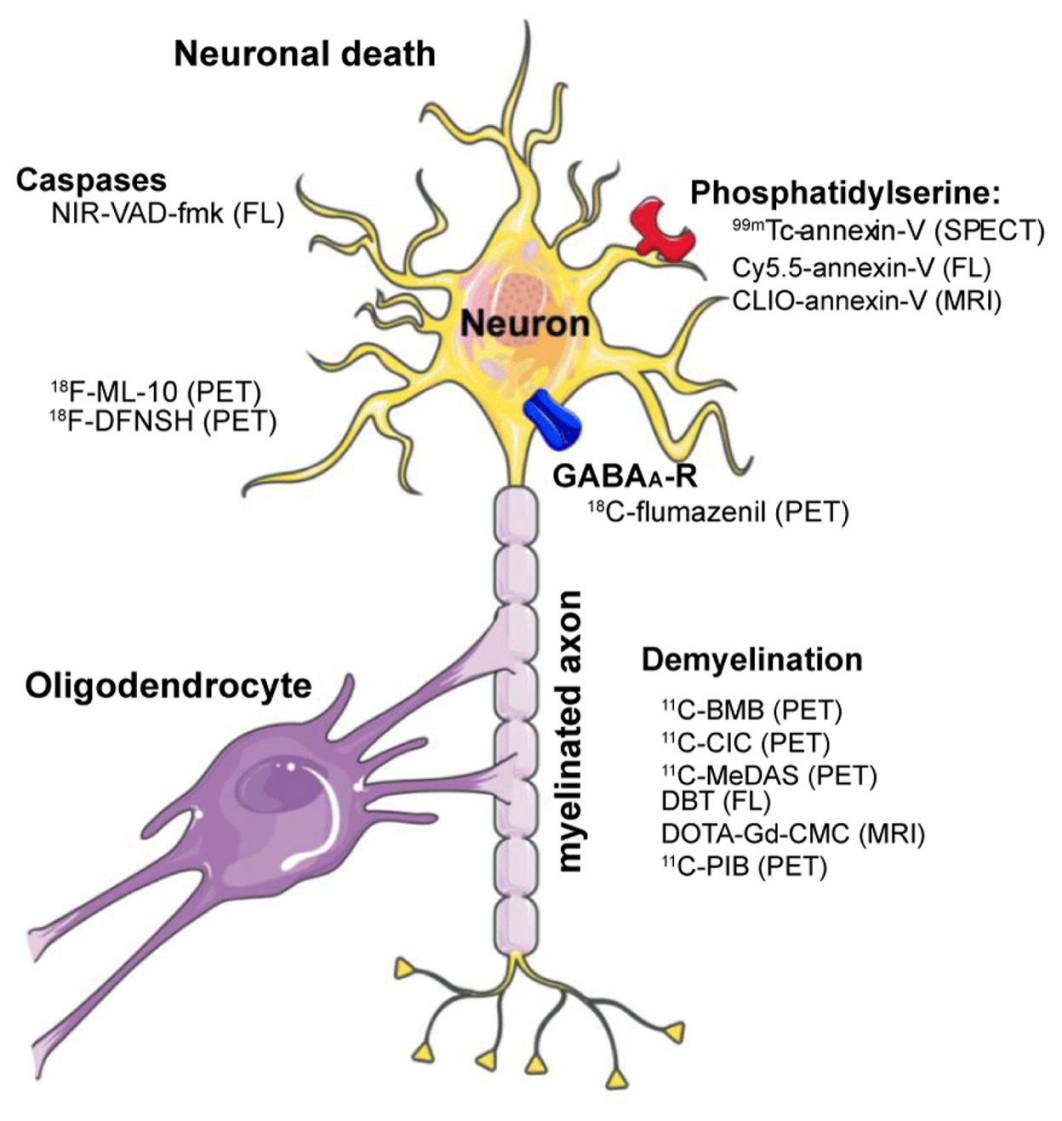
Molecular imaging targets and probes to visualize damage caused by neuroinflammation. Neuronal damage and demyelination are hallmarks of neuroinflammation. Injured neurons express phophatidylserine, which can be targeted by annexin-V. Caspases are key mediators of neuronal apoptosis, while Aposense agents enter dying cells that have lost integrity of their membranes. GABAa-receptor expression has been established as a marker for neuronal integrity. The myelin sheath around axons is electrically insulating and allows for fast signal transmission. Several different agents have been demonstrated to specifically bind to myelin, thus allowing for assessment of demyelination in neuroinflammation. FL: Fluorescence imaging (Adapted from Servier Medical Art).

Molecular imaging techniques that non-invasively visualize specific targets of the inflammation cascade using specific and sensitive probes could be powerful tools to evaluate neuroinflammation in the clinical and pre-clinical settings. This could allow for more sensitive and earlier detection as well as for monitoring disease progression and response of patients to therapeutic interventions. Over the past decade, a plethora of molecular imaging agents have been developed and tested in animal models of neuroinflammation, and a few have been translated from bench to bedside. The most promising imaging techniques to visualize neuroinflammation include MRI, positron emission tomography (PET), single photon emission computed tomography (SPECT), and optical imaging methods. In this review, we focus on molecular imaging techniques that target inflammatory cells and molecules that are of interest in neuroinflammation, especially those with high translational potential. Figure 1 illustrates the targets and the imaging agents for neuroinflammation, while Figure 2 shows the targets and agents for damage caused by neuroinflammation.

## Adhesion Molecules

Migration of blood-borne leukocytes through the endothelium (and the BBB) is a multi-step process consisting of chemoattraction, adhesion, and transmigration [11,12]. While chemoattraction is mediated via various cytokines that often have redundant functions and cell targets, adhesion is mediated through interaction of endothelial cell selectins (e.g., P- or E-selectin), VCAM-1, or ICAM-1 with leukocyte integrins (e.g., VLA-4, LFA-1, Cd11b, etc.). Further interaction with adhesion molecules such as PECAM-1 facilitates extravasation into the subendothelial space, where exposure to the local cytokine microenvironment directs leukocytes further towards their target [12]. Drugs that target leukocyte adhesion molecules can be highly efficient, exemplified by the monoclonal antibody natalizumab, which binds α_4_β_1_ integrin and is an established drug for MS [13].

Molecular imaging probes to detect adhesion molecules must have several characteristics to be successful: First, their physical size has to be big enough (generally >500 kDa) to prevent non-specific leakage through a compromised BBB. Second, good binding capacity under conditions of high shear stress in vessels is important. Finally, high sensitivity and specificity is key to detect subtle changes in the expression of these molecules in inflammation.

### Vascular cell adhesion molecule-1 (VCAM-1 or CD106)

**Figure 3:**
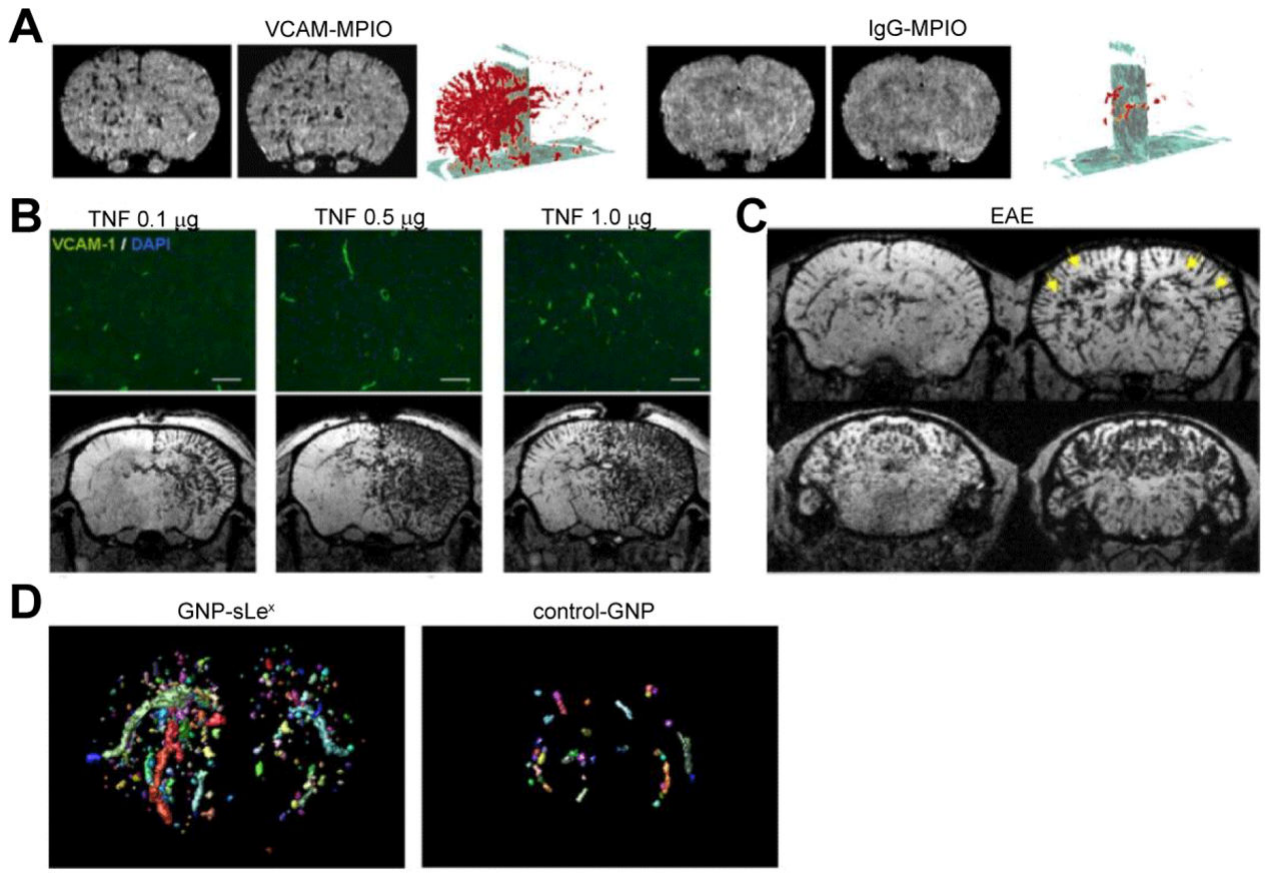
MR Imaging of adhesion molecules in neuroinflammation. (A) VCAM-MPIO compared to IgG-MPIO enhanced MR imaging after intracerebral injection of IL-1β, axial T2*-weighted images and 3-dimensional volumetric maps of VCAM-MPIO (or IgG-MPIO) binding are shown. (B) VCAM-upregulation after intracerebral injection of TNF on histology and VCAM-MPIO-enhanced MRI. (C) Diffuse VCAM upregulation in the brain of an EAE mouse on VCAM-MPIO-enhanced MRI. (D) 3D reconstruction maps of GNP-sLex- and control GNP-enhanced MRI in EAE mice reveal increased selectin expression in the inflamed brain. (Modified from McAteer etl al. [14], Montagne et al. [19], and Serres et al. [29] with permission).

VCAM-1 is an adhesion protein of the immunoglobulin superfamily expressed on endothelial cells. It is highly upregulated following stimulation with cytokines, and facilitates adhesion of different leukocyte populations. In a proof-of-concept study, McAteer et al. [14] conjugated an anti-VCAM-1 antibody to 1 μm sized micron particles of iron oxide (MPIO), further referred to as VCAM1-MPIO. In a mouse endothelial cell line stimulated with TNF to upregulate VCAM-1 expression, VCAM1-MPIO retention was detected. *In vivo*, after intracerebral injection of IL-1β into the striatum, focal hypointensities on T2 weighted MR imaging were detected consistent with accumulation of VCAM1-MPIO (Figure 3A). Specificity of this approach was demonstrated by injecting an isotype IgG antibody conjugated to MPIO (IgG-MPIO) and pre-treatment with an anti-VCAM-1 antibody to block binding of VCAM1-MPIO. In both experiments, no significant T2 signal alteration was detectable [14]. Applying the same probe to a mouse model of brain metastases using 4T1 or MDA231BR cell injections, VCAM1-MPIO allowed for earlier detection of metastases, and the authors concluded this might translate to improved detection of metastases [15]. In cerebral ischemia using the middle cerebral artery occlusion (MCAO) model, a greater area of VCAM-1 expression was detected compared to the DWI hyperintense area, suggesting that hypoperfused brain regions at risk for infarction upregulate VCAM-1 [16]. In experimental autoimmune encephalomyelitis (EAE), a mouse model of MS, VCAM1-MPIO allowed for detection of subclinical disease at a stage when lesions were undetectable by DTPA-Gd enhanced MR imaging. In symptomatic mice, VCAM1-MPIO detected all lesions visible with DTPA-Gd plus additional lesions corresponding to areas of leukocyte infiltration on histology [17]. Finally, utilizing the pilocarpin model of seizure, focal hypointensities were found in the periventricular organs, the hippocampus, and cerebral cortex with VCAM1-MPIO [18].

While these studies provide proof-of-principle evidence that molecular MR imaging targeting endothelial VCAM-1 expression in vivo is feasible, a major concern is sensitivity in diseases with only subtle inflammation, such as dementia. Montagne et al. [19] addressed this issue by using a different anti-VCAM-1 antibody clone. While McAteer et al. used M/K-2, Montagne et al. evaluated both M/K-2 and A(429), and found a greater than 250% signal increase with A(429). With this optimized agent, they were able to detect not only inflammation in EAE and after intracerebral TNF injection (Figure 3B–3C), but also in the unilateral common carotid artery occlusion model of vascular dementia, the APPPS1 model of Alzheimer disease, as well as subtle neuroinflammation after systemic challenges with LPS, ethanol, or glucose [19]. Extending their findings to stroke, the same group found extended VCAM-1 upregulation with permanent but not transient MCAO in peri-infarct areas, suggesting an inflammatory penumbra that subsequently infarcts [20].

Another approach to image VCAM-1 not yet applied to neuroinflammation includes affinity peptide ligands for MRI or PET/CT. In one study, phage display derived peptides were screened *in vitro* through a murine endothelial cell line to identify a candidate peptide that is internalized specifically via VCAM-1. This peptide was conjugated to CLIO-Cy5.5 for dual MR and fluorescence imaging of atherosclerosis [21]. *In vivo* phage display later identified a linear peptide affinity ligand named VINP-28, further refining this approach. VINP-28 is homologous to VLA-4, which is a known ligand for VCAM-1. After conjugation to CLIO-Cy5.5, affinity was found to be 20 times higher compared to the former approach, and dual modality imaging was performed with MR and fluorescence imaging in atherosclerosis [22]. A similar affinity peptide ligand was also labeled with 18F for PET/CT imaging, and successfully tested in mouse models of atherosclerosis, myocardial infarction, and cardiac transplant rejection [23].

### E-/P-selectin (CD62)

E- and P-selectins (or CD62E/P) are adhesion molecules expressed on endothelial cells, and they are also upregulated in the presence of inflammation. A binding partner for these molecules is sialyl Lewis^x^ (sLe^x^), which is expressed on leukocytes. Interaction between selectins and sLe^x^ mediates rolling adhesion of leukocytes alongside activated endothelial cells in inflammatory conditions. Fu et al. [24] synthesized a mimetic of sLe^x^ coupled to DTPA. The resulting Gd chelate, Gd-DTPA-B(sLe^X^)A was then tested in intracerebral injection of TNF and IL-1β, where a moderate increase in the MRI T_1_-weighted signal was seen at 50 minutes post contrast injection, while no difference was appreciated after injection of the nonspecific DTPA-Gd [25]. In the transient MCAO stroke model, the same agent was capable of detecting upregulation of E-/P-selectin in the infarcted brain region, again with relatively low sensitivity [26]. To address the issue of sensitivity, the same group developed MNP-PBP, a peptide ligand specific for P-selectin conjugated to a 50 nm diameter aminated dextran superparamagnetic iron oxide (SPIO) nanoparticle. This agent was again tested in the transient MCAO stroke model with improved but still suboptimal sensitivity [27].

To further increase sensitivity, a glyconanoparticle (GNP) reagent named GNP-sLe^x^ was designed to bind to E- and P-selectin. This nanoparticle bears 10^5^ to 10^7^ sLe^x^ moieties on the surface of an amine-functionalized dextran-coated ultra-small paramagnetic iron oxide (USPIO). Using an unmodified control nanoparticle as a comparison proved specificity of GNP-sLex. After intracerebral injection of IL-1β, upregulation of E-/P-selectin was detectable in the injected hemisphere. These results were further corroborated in focal MOG-induced EAE and endothelin-1 (ET-1) induced stroke in rats (intracerebral injections). In both disease models, T2 hypointense areas were detected consistent with upregulation of E-/P-selectin in neuroinflammation [28]. Importantly, control nanoparticle did not reveal any changes, and injection of DTPA-Gd did not show BBB alterations or changes in CBV, confirming specificity and subclinical detection capability of this approach. In a follow-up study, this agent was also used to evaluate subclinical focal EAE lesions after reactivation with systemic LPS, where GNP-sLex was a highly sensitive marker for subclinical inflammatory foci (Figure 3D) [29].

### Intercellular adhesion molecule 1 (ICAM-1, or CD54)

ICAM-1 is another endothelial cell expressed member of the immunoglobulin superfamily involved in adhesion of leukocytes to the endothelium. The first approach to perform MR imaging of an adhesion molecule, and in this case ICAM-1, was undertaken by Sipkins et al. [30], when they used an anti-ICAM-1 conjugated liposome chelate. Using *ex vivo* brain MR imaging after injection of their agent in the EAE model of MS, they were able to detect upregulation of ICAM-1 in the inflamed brain. Localization of this probe to the endothelium was confirmed with a Texas red tagged probe under fluorescence microscopy [30]. Subsequently, Deddens et al. [31] designed and tested two different probes targeted at ICAM-1. Their first probe, an anti-ICAM-1 functionalized Gd liposome (similar to the probe used by Sipkins et al. [30]) with a size of approximately 200 nm worked *in vitro* when tested in brain endothelial cells stimulated with TNF, but lacked sensitivity to detect ICAM-1 upregulation in a murine stroke model. In contrast, an anti-ICAM-1 functionalized MPIO (ICAM-MPIO) with a size of approximately 1 μm bound specifically to TNF-stimulated brain endothelial cells *in vitro*, and showed T2 hypointense brain areas 1 hour after induction of the transient MCAO stroke model *in vivo* [31]. In a subsequent study using the same agent, upregulation of ICAM-1 in the brain after radiation injury could be visualized [32].

### Integrin αvβ3

Integrin αvβ3, a cell adhesion molecule not only expressed on endothelial cell but also on macrophages and platelets, has also been targeted for non-invasive imaging. Peptides containing the three amino acid sequence arginine-glycine-aspartic acid, called RGD peptides, have been shown to be specific ligands for integrin αvβ3, and were labeled with radioactive tracers for PET and SPECT [33,34]. Two agents, ^18^F-galacto-RGB for PET and ^99m^Tc-NC100692 for SPECT have been translated [35], but human studies have been restricted to evaluation of tumor angiogenesis and not neuroinflammation. In addition to nuclear imaging, MR imaging with RGD peptides conjugated to Gd-based paramagnetic nanoparticles [36], and USPIO-based superparamagnetic nanoparticles [37] were used to assess tumor angiogenesis. Furthermore fluorescent molecular tomography (FMT) with RGD-peptides conjugated to Cy5.5 [38] and quantum dots [39] have been reported. In a mouse model of glioma, Cy5.5-RGD binding was evaluated on fluorescence reflectance imaging (FRI), and signal intensity correlated well with tumor size on MR imaging. Binding was blocked by pre-injection with unlabeled RGD, and Cy5.5 signal co-localized with vasculature on fluorescence microscopy [40]. Using a tetrameric ^64^Cu labeled RGD peptide, Wu et al. [41] similarly demonstrated rapid and significant probe uptake into mouse gliomas, and specificity was confirmed by pre-injection with unlabeled RGD to block binding sites.

## Leukocyte Functions

### Oxidative stress

#### Myeloperoxidase

Myeloperoxidase (MPO) is a proinflammatory and oxidative enzyme secreted by activated neutrophils and monocytes in inflamed tissues, facilitating the conversion of H_2_O_2_ to HOCl [42]. Querol et al. [43,44] synthesized the paramagnetic activatable MPO sensor Gd-bis-5-HT-DTPA (MPO-Gd). MPO oxidizes the sensor’s 5-hydroxytryptophan (5-HT) moieties to results in two effects: 1) oligomerization of the sensor and 2) binding of the sensor to proteins. Both effects lead to an increase in T_1_ relaxivity, and because the sensor is retained at sites of MPO activity, delayed image acquisition improves sensitivity and specificity [43]. Probe retention and biodistribution were investigated by labeling bis-5-HT-DTPA with ^111^In, and the MPO sensor was more than fourfold increased at MPO-rich sites [43].

**Figure 4:**
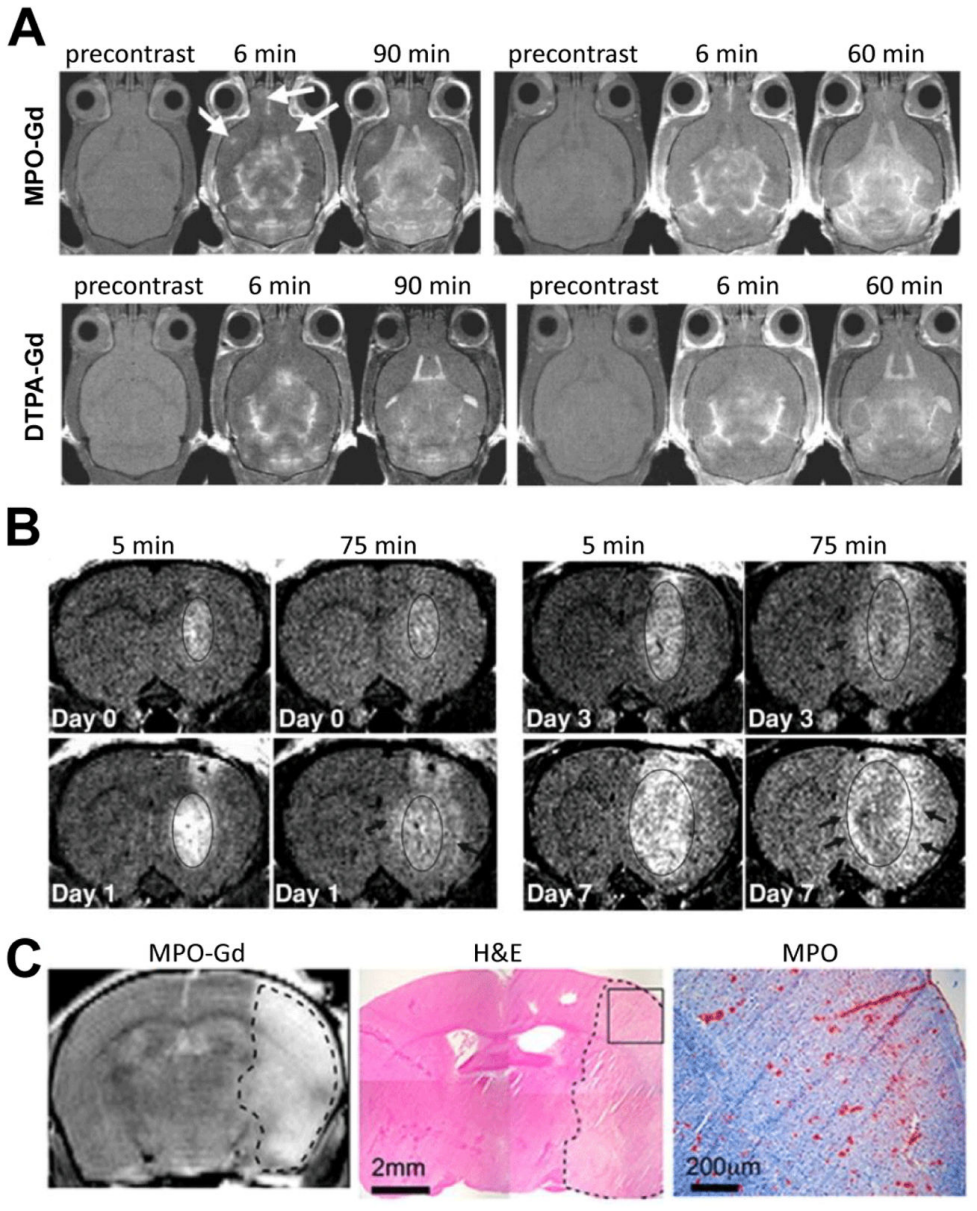
Molecular MR imaging of myeloperoxidase. (A) MPO-Gd enhanced MRI in EAE, where more lesions (arrows) are detected with MPO-Gd compared to DTPA-Gd. In addition, delayed enhancement confirms MPO-mediated activation. (B) MPO-Gd enhanced MRI in glioma shows low MPO activity before oncolytic virus administration (day 0). On days 1 and 3, MPO-Gd contrast increased in the peritumor area but is still present in the tumor center. On day 7, most of the enhancement in the center fades, but persistent MPO-Gd enhancement in seen in the periphery. (C) MPO-Gd enhanced MRI on day 3 after stroke correlates well with infarct on H&E staining on histology, where MPO immunostaining is present. (Modified from Chen et al. [45], Breckwoldt et al. [49], and Kleijn et al. [47] with permission.)

Since then, MPO-Gd has been used in several neuroinflammatory diseases. In EAE, MPO-Gd detected more and smaller active inflammatory brain lesions than DTPA-Gd (Figure 4A), and MPO-expressing cells and demyelinated areas correlated well with MPO-Gd enhanced MR imaging findings [45]. In a subsequent study, a preclinical MPO-inhibitor was found to significantly ameliorate clinical disease in EAE mice by reducing infiltrating inflammatory cells and demyelination, and MPO inhibition resulted in reduced lesion volume, lesion number, and enhancement intensity on MPO-Gd enhanced MR imaging [46]. These findings suggest that MPO could be a potential treatment target and imaging biomarker in MS.

In the MCAO mouse model of stroke, MPO-Gd enhanced MR imaging detected widespread secretion of MPO into the ischemic areas, and MPO-Gd positive lesion volume correlated well with infarct size (Figure 4C) [47]. MPO-specific signal peaked on day 3 after stroke, which was confirmed using *in vitro* MPO activity and RT-PCR assays [47]. In addition, specificity of MPO-Gd *in vivo* was confirmed by imaging MPO-knockout stroke mice, in which no specific MPO enhancement was detected [47]. In a mouse model of silent brain ischemia induced by intra-arterial injection of microbeads or fractionated clots, MPO-Gd positive brain areas co-localized with embolic material [48].

Gliomas are known to trigger inflammation, and one clinical problem is to distinguish recurrent growth from inflammation. Kleijn et al. [49] injected rats intracerebrally with D74/HveC glioma cells or mice with CT-2A cells, and treated the resulting gliomas with an oncolytic virus. MPO-Gd enhanced MRI detected the inflammatory changes induced by treatment with oncolytic virus longitudinally *in vivo*: On day 1 after virus injection, intratumoral MPO activity elevated. On days 3–7 after virus injection, while tumor size decreased and intratumoral MPO activity decreased, peritumor MPO activity increased (Figure 4B). Upon translation, this might allow differentiation of tumor from inflammation.

Another approach to detect oxidative stress generated by the MPO system is based on emission of chemiluminescence by oxidizable probes. While luminol has been found to specific to MPO [50], lucigenin requires NADPH oxidase for activation [51]. Luminol has been used to visualize oxidative stress *in vivo* in a mouse model of stroke [52] and in EAE [53]. Because tissue absorption and light scattering are an issue for translation to humans, a chemilumescence resonance energy transfer methodology was recently described, where luminol emitted light excites nanoparticles to emit far-red fluorescence, resulting in signal amplification and reduced tissue absorption [54].

#### Free radicals

To detect free radicals, the radical spin trap 5,5-dimethyl-1-pyrroline N-oxide (DMPO) was injected into mice followed by an antibody against DMPO conjugated to albumin-biotin-DTPA-Gd. This approach proved fruitful in a mouse model of diabetes, where *in vivo* MR imaging distinguished diabetic from control mice [55]. In mice with a mutant form of superoxide dismutase 1 (SOD1), which develop a disease resembling amyotrophic lateral sclerosis (ALS), DMPO imaging was also successful in detecting free radicals. There, T_1_ signal 120 minutes post agent injection was elevated compared to control mice, and probe retention in the spinal cord of ALS mice but not control mice was confirmed *ex vivo* with streptavidin-Cy3 (which binds to the biotinylated probe) on fluorescence microscopy [56]. This suggests successful detection of oxidative stress *in vivo* in a mouse model of ALS.

Electron paramagnetic resonance imaging (EPRI) in principle is capable of detecting unpaired electrons in free radicals, but because free radicals are short-lived and exist at very low concentrations *in vivo*, administration of imaging agents with unpaired electrons or precursors of such agents is necessary [57]. In the kainic acid model of epilepsy in rats, a BBB-permeable nitroxide radical was injected to evaluate the reduction capability of certain areas in the brain. In the hippocampus, the ability to reduce radical was diminished compared to other brain regions and to hippocampus of control animals [57], and these findings were confirmed in the FeCl_3_ seizure model [58]. Using an oxidizable precursor sensor, increased levels of oxidative stress were detected in hippocampus and striatum in kainic acid induced seizure [59]. In a mouse model of cerebral ischemia-reperfusion, nitroxide radical enhanced EPRI demonstrated impaired reduction ability in the ischemic brain [60]. With EPRI, one of the limitations is spatial resolution. Overhauser MR (OMR) imaging couples the sensitivity of EPRI with the spatial resolution of MRI by making use of the Overhauser effect [61]. In mice with cerebral ischemia-reperfusion, decreased reduction capacity was detected in the affected hemisphere with methoxycarbonly-PROXYL (a redox sensitive agent) on OMR imaging [62]. Similarly, in the 6-hydroxydopamine model of Parkinson’s disease, decreased reduction capacity was detected in the injected hemisphere [63].

#### Other approaches

Other approaches to image oxidative stress *in vivo* are less advanced, or have not been applied to study neuroinflammation. A PET agent named ^18^F-5-Fluoro-L-Aminosuberic Acid (^18^F-FASu) is taken up by a cystine/glutamate transporter that is upregulated when cells experience oxidative stress to make substrate available for the antioxidant glutathione. ^18^F-FASu has been validated *in vitro* and *in vivo* using mouse xenograft tumors [64]. Peroxy Caged Luciferin-1 (PCL-1) is a bioluminescent probe specific for H_2_O_2_. In the presence of firefly luciferase and H_2_O_2_, luciferin is released from PCL-1 and triggers bioluminescence. This system’s suitability for *in vivo* imaging has been demonstrated in a mouse model of prostate cancer [65]. Lastly, CePO_4_:Tb, Gd hollow nanoparticles have been described to form nanospheres in the presence of H_2_O_2_, thus making them potentially suitable for fluorescence and MR imaging [66], but have not been tested *in vivo*.

### Proteolytic activity

Proteases such as matrix metalloproteinases (MMPs), which are a family of zinc-dependent endopeptidases with over 25 members, as well as cathepsins, which are cysteine proteases with at least 12 members, are crucial mediators of tissue damage secreted mostly by microglia, astrocytes, and monocytes/macrophages. MMPs degrade extracellular matrix and are associated with excitotoxicity, neuronal damage [67], and opening of the BBB [68].

**Figure 5:**
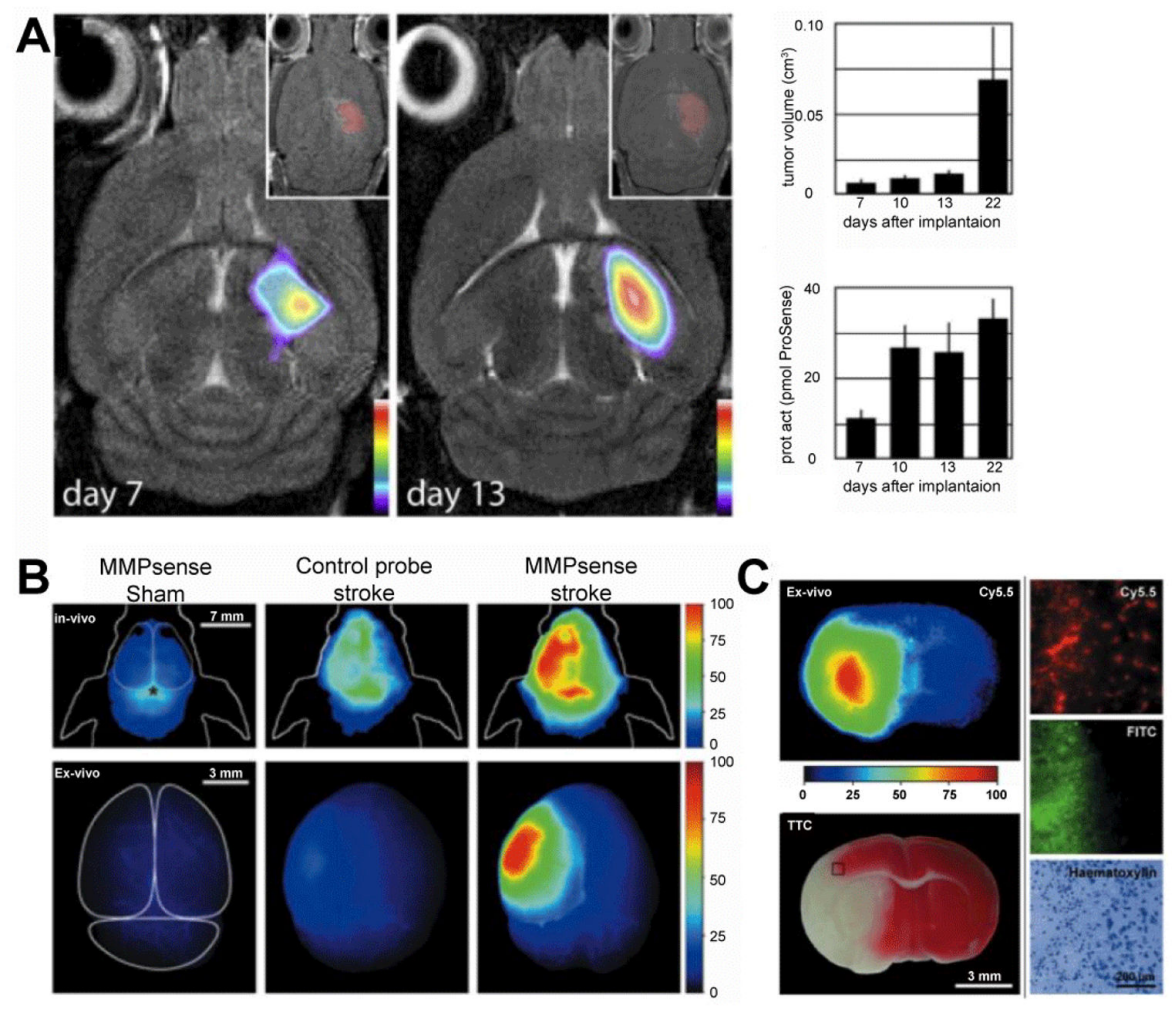
MMP imaging in neuroinflammation (A) Combined FMT and MR imaging of brain tumors with Prosense demonstrates focal activity of protease activity associated with gliomas. Prosense signal correlated well with tumor size on MRI. (B) In vivo fluorescence imaging at 24 hours after MCAO in mice, where strong fluorescence was detected over the infarcted hemisphere. In contradistinction, very little signal increase was seen with MMPsense in sham mice or with control probe in stroke mice [70]. (C) *Ex vivo* correlation of Prosense signal (Cy5.5 channel) with TTC staining, and with BBB breakdown (FITC-albumin) in stroke mice. (Modified from McCann et al. [72] and Klohs et al. [70] with permission.).

MMPsense is an activatable near-infrared fluorescence probe that can be cleaved by various MMP types [69]. MMPsense has been used visualize MMP activity in stroke, where increased MMP activity on FRI was detected in the ischemic brain (Figure 5B–5C) [70]. MMPsense signal was detectable at 24 hours, remained elevated until day 7, and was inhibited by a preclinical MMP inhibitor [70], suggesting specificity of this probe.

Prosense is a similar activatable near-infrared fluorescence probe that can be cleaved by cathepsin, B, L, and S. It was first evaluated in the 9L gliosarcoma mouse model in the brain, where elevated fluorescence signal was detected using FMT [71]. The same probe was also used in a study utilizing nude mice implanted with U87 human glioma cells. FMT was fused with MR imaging by use of landmarks such as ears, eyes, snout, and tumor foci for better anatomical coregistration. Prosense signal location and intensity correlated well with tumor location and growth (Figure 5A), and the response to chemotherapy with temozolomide could be visualized [72]. A fluorescent sensor specific to cathepsin B (Cath B 680 FAST) was successfully used in mice with EAE, where it distinguished EAE from control mice and demonstrated elevated cathepsin B activity in brain and spinal cord [73].

For nuclear imaging of MMPs, the MMP-3 inhibitor CGS 27023A has been radiolabeled with fluorine-18 (^18^F) for potential PET imaging [74]. An iodine-123 (^123^I) ligand of the same compound has been used to image vascular inflammation after carotid artery ligation with SPECT [75].

Other approaches include a radiolabeled antibody against MMP-14, which has been used to image inflammation in atherosclerotic ApoE deficient mice with SPECT [76], and the PET probe copper-64 (^64^Cu)-DOTA-CTT, which is based on the peptide MMP-2/9 inhibitor CTT, but poor *in vivo* stability and low affinity have halted further studies [77].

### Cyclooxygenase (COX)-1 and 2

COX-1 and 2 are key enzymes that catalyze conversion of arachidonic acid to prostaglandins, which play crucial roles in inflammation. While generally COX-1 is thought of as constitutively expressed, COX-2 is often upregulated during inflammation. However, in the brain it has been suggested that microglia express COX-1 and upregulate this enzyme in various inflammatory diseases, while COX-2 is mostly expressed by neurons in a constitutive way [78].

To visualize COX-1 activity, the PET ligand 11C-ketoprofen was successfully tested for specificity in COX-1 and COX-2 knockout mice. After intracerebral injection of LPS in rats, increased uptake of ^11^C-ketoprofen was seen as early as 6 hours and returned to baseline by day 7. This correlated with presence of COX-1 but not COX-2 expressing microglia at the site of injection [79]. Consistent with these findings, a PET agent selective for COX-2 did not show significant changes in a rat model of HSV encephalitis [80], and a different COX-2 PET agent showed high uptake in the brain reflective of constitutive neuronal COX-2 expression [81].

### Glycoprotein IIb/IIIA (GPIIb/IIIa)

GPIIb/IIIa is an integrin complex found on platelets, and is important for platelet activation. In cerebral malaria, extensive damage to vascular endothelial cells and platelet thrombi are typical disease features, and related to platelet activation via GPIIb/IIIa. Von zur Muhlen et al. [82] have conjugated a single-chain antibody directed against the ligand-induced bindings sites (LIBS) of GPIIb/IIIa on platelets to MPIO. The resulting agent LIBS-MPIO specifically detects the LIBS epitope, which is only exposed upon platelet activation. In a mouse model of cerebral malaria, using LIBS-MPIO enhanced MR imaging, they were able to detect activated platelets in the brain vasculature at a stage when conventional MR imaging was negative and clinical findings absent.

### Translocator protein (TSPO)

**Figure 6:**
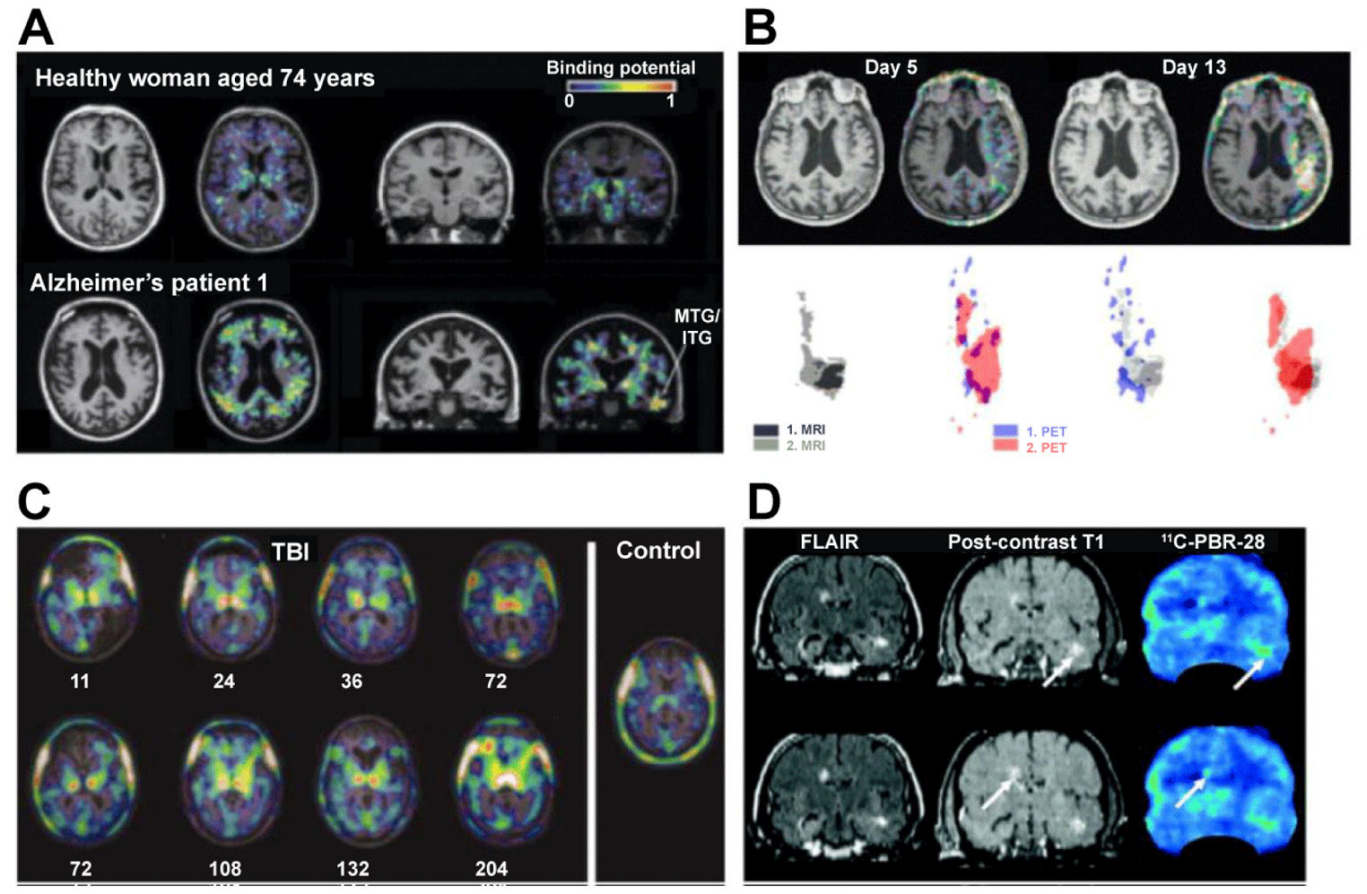
TSPO PET imaging in neuroinflammation. (A) 11C-PK11195 PET in patients with Alzheimer’s disease shows increased uptake suggestive of microglia activation in the entorhinal, temporoparietal and cingulate cortex. (B) ^11^C-PK11195 PET in a patient with acute ischemic stroke. Coregistered T1-weighted MRI and PET demonstrated ^11^C-PK11195 enhancement surrounding the infarct seen on MRI on day 5, while considerable overlap between MRI and PET was found on day 13. (C) Chronic microglia activation following TBI as evaluated by ^11^C-PK11195 PET, where increased uptake in the thalami of all TBI subjects is seen compared to control individuals. (D) In a patient with MS, focally increased uptake of the TSPO PET agent ^11^C-PBR28 is seen in gadolinium enhancing and FLAIR hyperintense MRI lesions (arrows). (Modified from Cagnin et al. [90] Gerhard et al. [93], Ramlackhansingh et al. [92], and Oh et al. [124] with permission.)

TSPO (formerly peripheral benzodiazepine receptor) is not expressed on neurons but rather micro- and macroglial cells, and is upregulated with microglia and astrocyte activation in different neuroinflammatory conditions as measured with PK11195, a specific ligand for TSPO [83,84]. However, subsequent studies revealed that binding of PK11195 correlated better with the number of activated microglia/infiltrated monocytes than astrocytes in models of TBI and stroke [85,86]. In rats induced with stroke, carbon-11 (^11^C)-PK11195 PET revealed microglia activation/monocyte infiltration into the infarct [87] This was subsequently confirmed in a human stroke patient, where ^11^C-PK11195 enhanced PET showed increased uptake in the affected hemisphere 13 and 20 days after stroke [88]. Moreover, PK11195 binding co-localized with activated microglia in a mouse model of Alzheimer’s disease [89]. Investigating microglia activation with ^11^C-PK11195 in 8 patients with Alzheimer’s disease, increased ^11^C-PK11195 uptake was observed in the entorhinal, temporoparietal and cingulate cortex (Figure 6A) [90].

Since then, ^11^C-PK11195 PET has been used in a variety of neuroinflammatory diseases both in rodents and humans. In a mouse model of TBI, prolonged microglia activation was detected 10 days after the insult [91]. In 10 patients with TBI, there was no increased binding of ^11^C-PK11195 PET at the original site of focal brain injury, but in the thalamus, putamen, internal capsule, and occipital cortices (Figure 6C). Additionally, microglia activation could be detected even 17 years after TBI, suggesting a potential for therapeutic intervention even at this time point [92].

In a study of 6 stroke patients, microglia activation was observed as early as day 3 post stroke, first at the outer rim of the infarct and spreading to the core (Figure 6B), but was also seen in distant brain areas consistent with Wallerian degeneration [93]. In 18 stroke patients imaged between 2 weeks and 6 months post symptom onset, microglia activation in the infarct was initially elevated, normalized over the study period, but– if prolonged – correlated negatively with clinical outcome. In addition to microglia in the infarct, there was prolonged microglia activation along the damaged pyramidal tract (PD) in the brainstem in patients where the PD was affected, and this correlated positively with clinical outcome [94]. These results support the hypothesis that microglia have both neurotoxic and neuroprotective functions depending on location and timing.

In a study on 12 patients with MS, microglia activation was not only detected in areas correlating with lesions detected on conventional MRI, but also in normal-appearing areas including cortical areas [95]. Furthermore, ^11^C-PK11195 binding in cortical areas correlated with clinical disability in a study of 18 RR and SP-MS patients [96]. In a PET-MRI correlation study on 22 MS patients, ^11^C-PK11195 binding was increased in DTPA-Gd enhancing lesions but was decreased in T_2_-lesions. However, during a relapse, an increase in normal-appearing white matter as well as transient increased uptake in T_2_-lesions was seen [97]. Lastly, ^11^C-PK11195 uptake in normal-appearing white matter increased with the degree of brain atrophy [98]. These results suggest that microglia activation is present outside of MRI-detected lesions, and that microglia activation contributes to both relapses and chronic progression in MS.

In two patients with Rasmussen’s encephalitis, PET imaging with ^11^C-PK11195 showed diffuse and focal increase in signal throughout the affected hemisphere consistent with postmortem neuropathological studies of diffuse microglia activation [99]. Microglia activation was also detected in the brains of patients with Parkinson’s disease and ALS [100,101], in spinal cords of rats after sciatic nerve injury [102], and in striatum of patients with Huntington’s disease [103].

Although ^11^C-PK11195 has been widely used in both animal and human research, it suffers several set-backs: It is associated with a high level of nonspecific binding, and has a poor signal-to-noise ratio [104,105]. In addition, ^11^C has a very short half-life of only 20 minutes which also negatively affects widespread clinical use. Therefore, several alternative TSPO agents have been synthesized [106].

Several second generation TSPO agents were tested in neuroinflammatory models on *ex vivo* autoradiography: The benzodiazepine Ro5-4864 was radiolabeled with ^125^I and evaluated in the rat C6 glioma model, with increased probe uptake in the tumor [107]. ^18^F labeled PK14105 and ^11^C labeled AC-5216 were compared to PK11195 in rodents intracerebrally injected with kainic acid, and demonstrated similar characteristics [108,109]. Price et al. [110] tested three compounds in a rat model of Huntington’s disease, but the compound with the highest contrast over normal-appearing brain had similar characteristics as ^11^C-PK11195. ^123^I-CLINDE was used in rat EAE and the cuprizone mouse model of demyelination [111,112].

Most promising are agents that have been directly compared to ^11^C-PK11195 in *in vivo* PET studies, or have been conjugated with radiolabels that have better characteristics than 11C, such as ^18^F. For example, ^11^C-DAA1106 showed specific binding 5 times higher than ^11^C-PK11195 in rodent models of Parkinson’s disease and TBI [113,114]. A slightly improved tracer, ^18^F-fluorethyl-DAA1106 was capable of visualizing inflammation in APP23 Alzheimer’s mice [115]. However, in patients, no difference was detected between Alzheimer’s patients and control patients [116], or between MS patients and control patients [117], suggesting different receptor-ligand interaction depending on the species.

After intracerebral injection of the excitotoxin AMPA, the tracer ^11^C-CLINME identified inflamed brain tissue with higher binding potential compared to PK11195 [118]. ^18^F-PBR111, the fluorinated version of ^11^C-CLINME, has been used to image acute and chronic EAE [119], and is currently undergoing a clinical trial on MS patients. ^11^C-vinpocetine showed favorable characteristics compared to ^11^C-PK11195 in four patients with stroke [120], and the sensitivity of ^11^C-vinpocetine for activated microglia was confirmed in a study of 9 stroke patients [121]. The same agent detected elevated microglia activation in elderly compared to young subjects, but failed to detect a difference between elderly subjects and patients with Alzheimer’s disease [122]. ^11^C-PBR28 was successfully tested in the MCAO rat stroke model, and demonstrated microglia activation in the outer infarct rim [123]. In MS patients, ^11^C-PBR28 binding was seen in active inflammatory lesions confirmed with contrast-enhanced MR imaging (Figure 6D), and sometimes preceded appearance of MRI lesions [124]. Other agents include ^18^F-DPA714, which has been tested in EAE and rodent herpes encephalitis [125,126], and demonstrated higher contrast-to-noise ratios than ^11^C-PK11195 in a murine stroke [127].

Challenges in optimizing TSPO agents for human imaging include interspecies variations in affinity of different agents, which makes extrapolation of rodent studies difficult to impossible, as well as recent discovery of a polymorphism in the TSPO gene that determines binding affinity to TSPO, which affects all agents but ^11^C-PK11195 [128].

### Cannabinoid receptor 2 (CB2)

While the cannabinoid receptor 1 is constitutively expressed in a variety of cell types, CB2 is thought to be expressed on microglia/macrophages and is upregulated with activation of these cells [129]. Horti et al. [130] evaluated ^11^C-A836339, a selective CB2 agonist, as a candidate probe for neuroinflammation and specifically for activated microglia. They found increased uptake with LPS-induced neuroinflammation and in the transgenic APPswe mouse model of Alzheimer’s disease. A similar probe, ^11^C-KD2 showed increased uptake in spinal cord sections from ALS patients on autoradiography [131]. However, another probe, ^11^C-NE40 did not detect a difference between stroke and sham-operated rats in the photothrombotic stroke model [132]. Lastly, a near-infrared probe selective for CB2 has been developed and successfully tested in a mouse tumor model [133]. The major challenge for CB2 imaging is specificity of CB2 for microglia/macrophages, because in neuroinflammation CB2 can be detected on T-lymphocytes, astrocytes, and microglia [134].

## Leukocyte Labeling

### Radiolabels

**Figure 7:**
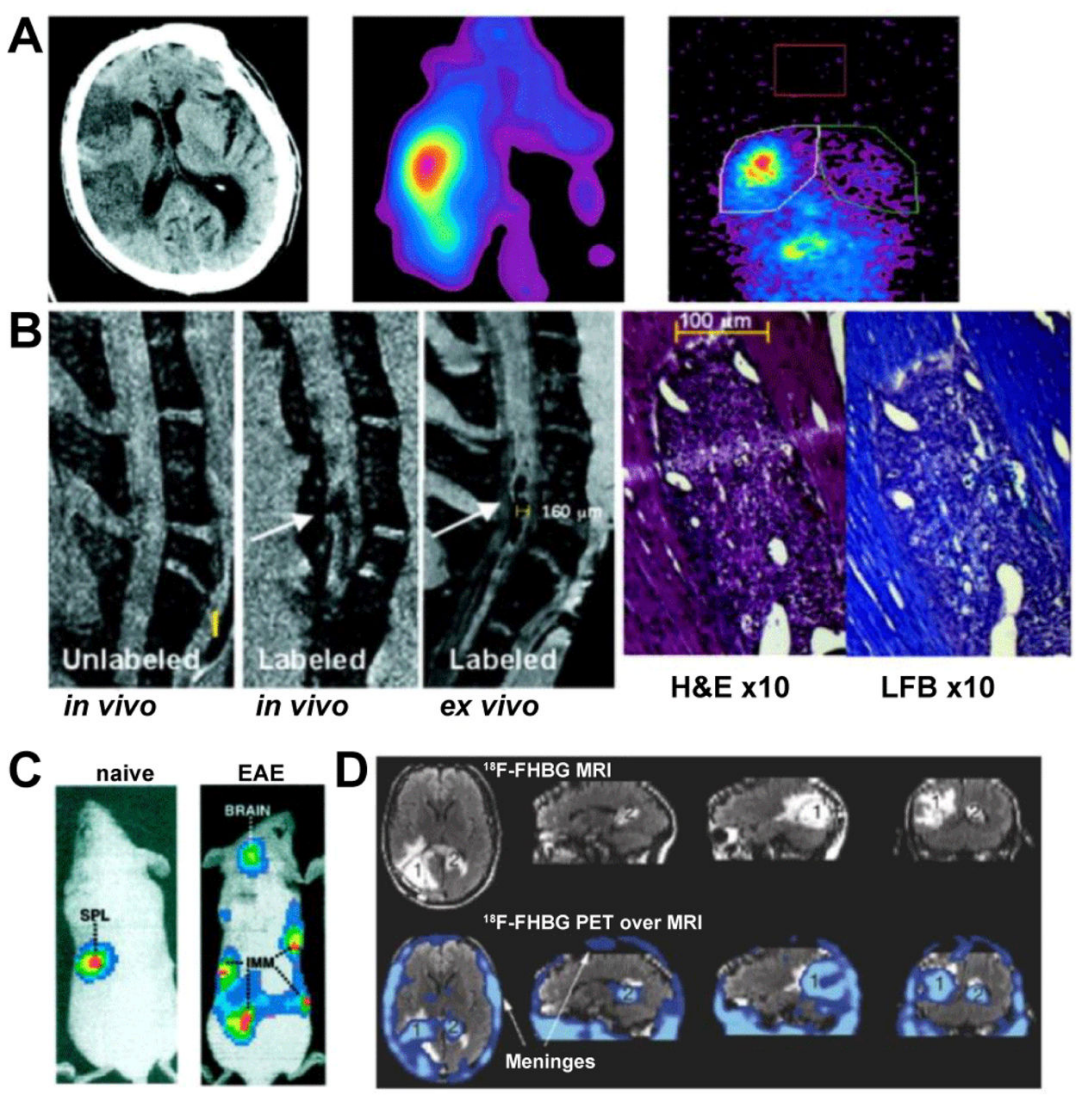
Cell tracking strategies. (A) Noncontrast CT in acute ischemic stroke and SPECT of *in vitro* ^111^In-troponolate-labeled neutrophils demonstrate cell infiltration into the infarcted brain region. (B) *In vivo* and *ex vivo* MR imaging of iron oxide labeled T-lymphocytes in EAE mice, and corresponding histological evidence of demyelination (H&E and luxol fast blue staining). (C) MBP-specific CD4^+^ T-lymphocytes were transduced with a GFP-luciferase retroviral vector and transferred into naïve or MBP-immunized EAE mice. On bioluminescence imaging, cells localized at the immunization sites as well as the brain in a clinically symptomatic mouse but not in a naïve control mouse. (D) MRI and PET of a patient with glioma injected with genetically targeted autologous cytolytic CD8^+^ T lymphocytes to express the IL-13 zetakine gene and herpes simplex virus 1 thymidine kinase suicide gene, which allows for PET detection using the radiotracer ^18^F-FHBG. (Modified from Price et al. [146], Anderson et al. [155], Costa et al. [205] and Yaghoubi et al. [206] with permission).

Decades ago, leukocytes were harvested from blood samples, labeled with indium-111 (^111^In) and re-injected to localize inflammation on SPECT. While first studies were mostly on sepsis foci detection [135–137], further studies extended the use of this approach to cerebral abscesses [138], brain metastases [139], and stroke [140]. However, ^111^In labeling damages leukocytes and this results in reduced proliferative capacity and DNA damage [141]. To circumvent this, technetium-99m (^99m^Tc) has been used, which carries a much lower risk of leukocyte damage [142]. In stroke, patients injected with ^99m^Tc-labeled leukocytes with poor outcome had higher signal in the affected hemisphere compared to patients with good outcome, suggesting a clinically relevant effect of leukocyte infiltration [143]. Wang et al. [144] demonstrated that ^99m^Tc-leukocyte signal is higher in acute than chronic stroke and that signal persists for several weeks. In a larger study on 88 stroke patients, leukocytes accumulated in infarcted brain areas and this correlated with brain tissue damage and poor neurological outcome [145]. To specifically track neutrophils, Price et al. [146] labeled these cells *in vitro* with ^111^In-troponolate. In 15 stroke patients, they found that neutrophils recruit to the infarcted brain within 24 hours (Figure 7A), and degree of neutrophil recruitment correlated with infarct extension [146].

Since then, this method has been adapted for PET imaging by labeling autologous cells with ^18^F-FDG and re-injecting them into patients. In a pilot study on 21 patients with fever of unknown etiology or unknown extent of infection, PET/CT imaging demonstrated general feasibility [147]. Additionally, labeling of leukocytes with ^64^Cu has been published [148].

However, there are several shortcomings of this approach: first, release of the radiolabel from the cells, which can penetrate a compromised BBB, leads to non-specific signal [149]; second, intraparenchymal versus intravascular signal cannot be distinguished [149]; third, false positive (e.g., GI bleeding, pseudoaneurysms, and tumors) and false negative (encapsulated nonpyogenic abscess, chronic low-grade infection, hyperglycemia, steroids) results are not uncommon [150]. Finally, length of follow up depends on the half-life of the radiolabel and on the proliferative capacity of the labeled cells (dilution of label per cell with signal loss).

### Iron oxide particles

Superparamagnetic iron oxide particles consist of an iron-oxide core embedded in dextran, citrate, or polymer shell, and several different particle sizes exist: ultra-small superparamagnetic iron oxide (USPIO, 10 – 50 nm), superparamagnetic iron oxide (SPIO, 50 – 100 nm), and micrometer-sized iron oxide (MPIO, >1 μm) [151]. These agents possess large negative magnetic susceptibilities, resulting in negative contrast on T_2_-weighted sequences. Virtually any cell type can be labeled *in vitro* with different-sized iron oxide nanoparticles, although for cells that do not phagocytose a transfection agent might be needed to increase iron oxide uptake [151].

Two main approaches in how iron oxide particles are used to image neuroinflammation exist: in the first, iron oxide is injected intravenously and taken up by phagocytic cells (e.g. monocytes, macrophages, microglia) *in vivo*, which are thus labeled, and the second, where autologous cells are harvested and labeled *in vitro*, then re-injected to trace the labeled cells.

#### Iron oxide labeling in vitro

In one of the few studies on iron oxide leukocyte labeling in neuroinflammation, Stroh et al. [152] isolated splenic mononuclear cells and labeled them with USPIO before systemic reinjection into stroke mice. They were able to detect injected cells at 48 hours in the lesion border for up to 5 weeks post stroke [152]. In a proof-of-concept study, human monocytes were labeled with superparamagnetic iron oxide (SPIO, Feridex, size 120–160 nm), which were injected into the basal ganglia of SCID mice. The SPIO nanoparticles were tracked by MRI for up to 14 days after injection, and MR imaging localization of monocytes was confirmed on histology [153].

Yeh et al. [154] labeled rat T-lymphocytes *in vitro* with USPIO, which took up by these cells via endocytosis, and reinjected them into the same rats for tracking by MRI. Anderson et al. [155] used SPIO-poly-L-lysine to label T-lymphocytes *in vitro*, before stimulation and adoptive transfer of these cells into recipient mice to induce EAE. SPIO-labeled T-lymphocytes were readily detected in the spinal cord of mice on *in vivo* and *ex vivo* MRI (Figure 7B) [155]. Using a similar approach, T-lymphocytes were labeled with SPIO in the presence of the transfection reagent protamine sulfate, which increased labeling efficiency and also allowed for detection of injected cells in the spinal cord on MRI [156].

Iron oxide particle labeling *in vitro* shares similar issues with radiolabeling. Cells might lose the label or die, both of which will result in nonspecific signal, and cell proliferation will dilute the signal. Both effects pose a limit on the time window of imaging. For clinical safety and validity of study results, any effects of iron oxide particles on their target cells need to be addressed. In one *in vitro* study, iron oxide nanoparticles did not affect proliferative capacity of a human macrophage cell line but transiently increased oxidative stress [157], consistent with phagocytic oxidative burst after phagocytosis of these particles. In addition, phagocytosis of SPIO or USPIO shifted rat and mouse macrophages towards an anti-inflammatory phenotype [158].

Another limitation is the restriction to one label with MR imaging (in contrast to fluorescence imaging, which can distinguish labels based on excitation and emission wavelength). Thus, cell co-localization and interaction cannot be investigated with conventional MR imaging and gadolinium or iron oxide based contrast agents. Recently, however, an interesting approach utilizing paramagnetic chemical exchange saturation transfer agents has been reported. There, lanthanides such as Yb and Eu are conjugated to HPDO3A, and these agents have similar pharmacokinetic and biosafety properties as Gd-HPDO3A. Using these agents, two different cell lines were labeled and could easily be distinguished *in vitro* as well as *in vivo* after injection into mice [159].

#### Phagocytosis/uptake in vivo

The first study using iron oxide to track cells that take up iron oxide particles via phago- or endocytosis *in vivo* was in a rat glioma model using C6 cells. C6 cells are known to phagocytose, and uptake of monocrystalline iron oxide nanoparticles (MION) of 20 nm diameter was shown *in vitro* by radionuclide and fluorescent labeling of MION. *In vivo*, gliomas showed uptake of MION on MRI [160]. In a human study involving 55 patients, in 19 of 34 glial tumors iron oxide enhanced as much or more than DTPA-Gd [161]. Taschner et al. [162] reported USPIO enhancement in 7 of 9 glioma patients, but only detected USPIO in macrophages in 2 patients, casting doubt on the cellular identity imaged with this approach.

For most other neuroinflammatory diseases, however, the ability of certain leukocytes (macrophages, neutrophils, monocytes, microglia, dendritic cells) to phagocytose iron oxide nanoparticles has been used in an attempt to specifically visualize these cells.

**Figure 8:**
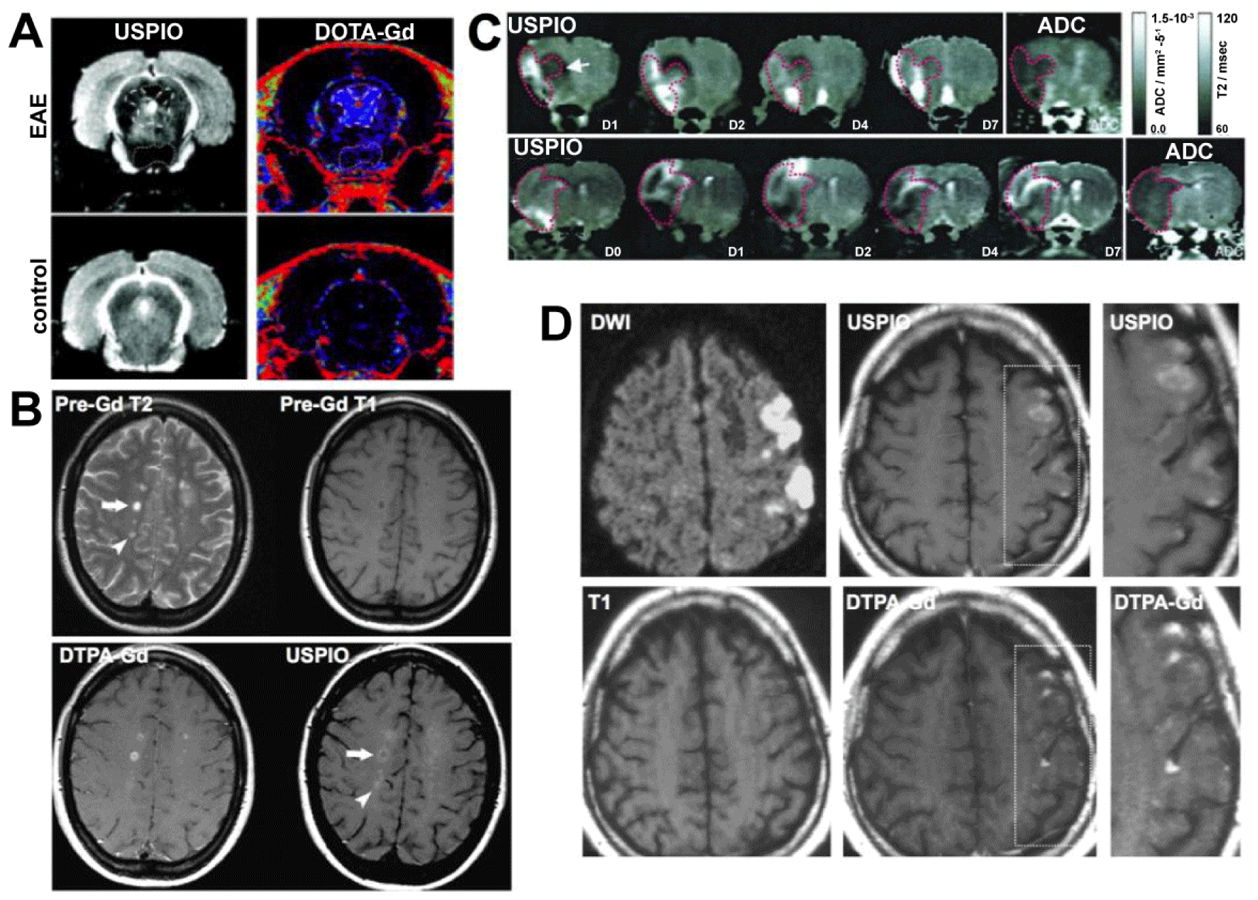
Imaging of phagocytic cells with iron oxide-enhanced MR imaging. (A) USPIO and DOTA-Gd-enhanced MR imaging of EAE rats. A periventricular lesion shows DOTA-Gd enhancement but not USPIO uptake. Vice versa, a ventral lesion shows USPIO uptake but no DOTA-Gd enhancement. No enhancement is seen in a control animal. (B) Pre-Gd T2 MRI on a MS patient demonstrates multiple lesions, and while there is a DTPA-Gd and USPIO enhancing lesions (arrow), there also is an USPIO-uptake-only lesion (arrowhead). (C) Spatiotemporal profile of USPIO uptake in the ischemic rat brain over 7 days. While USPIO uptake is first seen in the periphery of the infarct (ADC map), uptake becomes more central on the following days, while no uptake is seen on day 7. (D) Comparison of USPIO and DTPA-Gd in a patient with ischemic stroke. Diffusion-weighted imaging displays ischemic area. Areas of USPIO uptake are clearly different from gadolinium enhancement. (Modified from Rausch et al. [10,171], Vellinga et al. [166] and Saleh et al. [173] with permission.)

In the rat EAE model, USPIO-enhanced MR imaging, lesions of low T_2_ signal were seen and histologically confirmed to be phagocytic leukocytes [163]. When comparing USPIO enhancement (phagocytes) to DOTA-Gd enhancement (BBB breakdown), uptake of USPIO was detected in areas without DOTA-Gd enhancement, and vice versa (Figure 8A) [10]. In addition, USPIO enhancing lesions had low magnetization transfer ratios, possibly indicating co-localization of phagocytic cells with areas of demyelination [10]. USPIO lesion load and volume at the acute EAE stage correlated with inflammation, phagocyte infiltration, demyelination, axonal damage and extent of axonal loss [164].

In a study investigating BBB breakdown with DTPA-Gd and phagocyte infiltration with USPIO suggested that BBB breakdown preceded phagocyte infiltration in acute EAE [165]. In 19 patients with relapsing-remitting (RR)MS, who underwent serial MR imaging with USPIO and DTPA-Gd, 188 USPIO positive lesions were seen, 144 of which did not show DTPA-Gd enhancement. Of 59 lesions with DTPA-Gd enhancement, 15 did not show USPIO uptake, and USPIO uptake preceded DTPA-Gd enhancement in some lesions (Figure 8B) [166]. These results suggests that BBB breakdown and phagocyte infiltration are different pathophysiological events and might occur independently of each other at different stages or types of MS lesions, with no clear temporal relationship.

In the transient MCAO stroke model in rats, USPIO could be detected in vessels 24 hours after administration, and in infarcted tissue on day 2, consistent with neutrophil and monocyte infiltration at this time [167]. Localization of USPIO within phagocytic cells was confirmed on histology [168]. Wiart et al. [169] demonstrated peri-infarct USPIO uptake followed by signal spread to the contralateral hemisphere, correlating with areas of inflammation on histology. In a rat model of cerebral ischemia-reperfusion, SPIO uptake was mostly seen in the damaged brain and corresponded to areas of macrophages infiltration on histology [170]. To further clarify the timing of phagocytosis and possible cell migration, USPIO was injected on day 0 and uptake in stroke was investigated from days 1–7 after permanent MCAO in rats, and the following pattern was found: first, phagocytic cells accumulated in the boundary zone, then they were found in the infarct core on days 2–4, before clearing by day 7 (Figure 8C) [171]. This raised the question whether these cells could migrate into the ischemic lesion from the periphery. To address this question, the photothrombotic stroke model in rats, which yields highly reproducible infarcts without surrounding penumbra, was utilized. MR imaging was performed on days 3, 6, 8, and 14 post stroke induction, and 24 hours after injection of USPIO. On day 6, the outer infarct rim showed USPIO uptake, while by day 8 the infarct core was T_2_ hypointense [172]. In addition, there was BBB breakdown as evidenced by DTPA-Gd enhancement at all time points, indicating that BBB breakdown and phagocyte infiltration are unrelated [172]. Interestingly, when USPIO were injected on day 5, an outer rim of USPIO uptake was detected on days 6 and 8, indicating that phagocytes remain sessile [172]. Importantly, while macrophages were still found in infarcted tissue on day 14, no USPIO uptake was detected [172], suggesting a switch toward a non-phagocytic phenotype.

In a study on 10 ischemic stroke patients injected with USPIO 7 days after symptoms onset, USPIO uptake was seen in ischemic brain area and differed from DTPA-Gd indicated BBB breakdown, showing that phagocyte imaging is feasible in stroke patients (Figure 8D) [173]. In a second study of 10 stroke patients, USPIO volume did not correlate with DWI volume or BBB disruption, also suggesting that iron oxide MR imaging could provide information in addition to infarct size [174].

A central question when tracking phagocytic cells in the brain with iron oxide particles is when cells took up the particles. The first possibility is that circulating leukocytes phagocytosed particles in the blood stream and subsequently infiltrate into the brain. The second is that already infiltrated or resident microglia phagocytosed particles in the brain *in situ*. In the latter case, nonspecific signal from passage of particles through an impaired BBB can be problematic. Disrupting the BBB using the freezing lesion model, dextran-coated iron oxide (approximately 50 nm in size) was detected in endothelial cells on electron microscopy after 1 hour and persisted for 4–8 hours. On MRI, iron oxide enhancement was seen consistent with BBB breakdown [175]. After opening the BBB with mannitol, both MION (size 20 nm) and SPIO (size 200 nm) led to enhancement in the entire hemisphere. While MION was detected in the intercellular space, SPIO were found in endothelial cells but did no cross the basement membrane [176]. These findings indicate that the size of iron oxide particles may determine passage through an impaired BBB. In an experiment using crush injury of the sciatic and optic nerve, Bendszus et al. [177] detected USPIO uptake in sciatic nerve where they also found monocyte infiltration, but did not detect USPIO uptake in the optic nerve where they found microglia activation without monocyte infiltration. These findings suggest that phagocytes take up iron oxide in the blood en route to the brain. However, a study comparing USPIO injection versus re-injection of *in vitro* SPIO-labeled monocytes in photothrombotic stroke found that contrast enhancement was markedly different. While USPIO enhancement was seen within hours, SPIO labeled monocytes were not detected until after 72 hours, suggesting that USPIO does not represent signals from monocytes labeled en route to the brain but rather nonspecific penetration of the BBB or endothelial cell uptake [178]. Therefore, caution and careful validation need to be performed when tracking cell using parenterally injected iron oxide nanoparticles. *In vitro* labeling of the cells prior to injection should be preferred to avoid nonspecific signal.

Another potential issue with iron oxide phagocytosis is that phagocytosis per se does not differentiate between tissue damage and repair, as pro-inflammatory cells as well as anti-inflammatory cell can phagocytose. In human monocytes, it has been shown that CD14^++^ CD16^-^ classic (proinflammatory) monocytes phagocytose significantly more iron oxide than CD14^++^ CD16^+^ non-classical (anti-inflammatory) monocytes, as tested with three different SPIO agents (Ferumoxides, Ferucarbotran, CLIO) and one MION agent (MPIO-48) *in vitro* [179]. However, both monocyte subsets did take up all four particle types. This distinction is of clinical relevance, because in MS, macrophages that have phagocytosed myelin have been shown to be of the anti-inflammatory (repair) M2 phenotype [180]. Thus, one needs to corroborate the imaging findings against other datasets to ascertain the type of the cells imaged.

### Perfluorocarbons 19F-MRI, Gadofluorine M

Another approach to phagocytic cell labeling is fluorine ^19^F-MR imaging. In stroke mice injected with nanoemulsions of perfluorocarbons increased (^19^F) MR signal was detected in the border zone of ischemia, and these areas were confirmed to contain phagocytic leukocytes on histology [181]. Gadofluorine M is a gadolinium based agent with a perfluorinated side chain which forms small aggregates and micelles in solution approximately 5 nm in diameter [182]. Phagocytes and endothelial cells have been shown to take up this agent, and in a study on EAE, more lesions were detected compared to DTPA-Gd, with signal corresponding to areas of inflammation and demyelination [183]. Gadofluorine M also can be used to label cells *in vitro*, as shown with monocytes that could be detected on MR imaging for up to 7 days [184]. Little specificity data is available for these agents for phagocytosis imaging. However, given the size of these two agents, they may share similar limitations as USPIO nanoparticles regarding cell phenotype and nonspecific leakage.

### Specific cell subset labeling

Neutrophils: Neutrophils was first imaged with SPECT using ^99m^Tc-anti-CD15 (LeuTech) [185,186] an anti-granulocyte fab fragment of anti-NCA-90 conjugated to ^99m^Tc (LeukoScan) [187], or BW 250/183 anti-granulocyte antibody [188]. Another approach is to use formyl peptides, which comprise of a large group of heterogeneous molecules (e.g. Lipoxin A4, fMLF from *E.coli*, humanin) that bind to the formyl peptide receptor (FPR) group. FRP1 is mostly expressed on neutrophils and mediates migration to the site of inflammation [189]. ^111^In or ^99m^Tc labeled synthetic fMLF was successfully used to detect bacterial infection in experimental models [190,191], but it has been known to cause neutrophil activation and degranulation, making these compounds less desirable as imaging agents. The peptide cFLFLF is a high affinity antagonist for FRP1 and has been labeled with ^64^Cu resulting in the PET agent ^64^CU-PEG-cFLFLFK. This agent has been used to image lung inflammation [192]. The same peptide has been labeled with ^99m^Tc for SPECT, Cy7 for fluorescence imaging, and gadolinium for MRI [193–195]. Neutrophils also had been imaged using radiolabeled IL-8 [196], which binds to the IL-8 receptor on neutrophils and is important for chemotaxis, and a radiolabeled peptide binding to PF4/CXCL-4 [197], another important chemoattractant for neutrophils. However, none of these neutrophil labeling agents have been used to image neuroinflammation. Thus, neutrophil imaging is an untapped area of research and translation for neuroinflammation.

#### T-lymphocytes

First approaches to T- lymphocytes imaging were radiolabeled antibodies against CD3 and CD4, which were used to detect models of rheumatoid arthritis [198,199]. Radiolabeled cytokines such as IL-1 and IL-2 for SPECT were also used to detect inflammation in rheumatoid arthritis [200] and other autoimmune diseases [201, 202]. Recently, IL-2 was radiolabeled for PET with ^18^F resulting in ^18^F-FB-IL2, which was able to detect activated T-lymphocytes injected into SCID mice [203]. Unfortunately, injecting even small doses of cytokines often results in intolerable side effects.

Using an optical imaging approach, Berger et al. [204] labeled T-lymphocytes *in vitro* with Cy5.5-Tat, and tracked these cells to the brains of EAE mice after intravenous injection using FRI. Refining this approach, Costa et al. [205] isolated MBP TCR-Tg CD4^+^ TH lymphocytes and genetically engineered them to express GFP and luciferase. After injection into EAE mice, cells were tracked with bioluminescence imaging, and CD4^+^ TH lymphocytes were found first at sites of immunization and within the brain a few days later, persisting up to 50 days (Figure 7C) [205].

In an elegant proof-of-concept study in one patient with glioma, Yaghoubi et al. [206] genetically engineered autologous cytolytic CD8^+^ T lymphocytes to express the IL-13 zetakine gene and herpes simplex virus 1 thymidine kinase suicide gene. The former facilitates targeting of these cells to glioma cells, while the activity of the latter can be imaging with PET using the radiotracer ^18^F-FHBG (Figure 7D) [206]. This approach allowed for selective PET imaging after injection of genetically engineered lymphocytes, and avoided cell label loss or dilution.

#### B-cells

^99m^Tc-labeled rituximab (anti-CD20) has been used to image B-cell infiltration in patients with RA, Sjogren’s syndrome, Behcet’s disease, and sarcoidosis, but not yet in neuroinflammation [207]. Similarly, radiolabeled anti-CD19 and CD22 antibodies have been reported for B-cell imaging [208,209].

#### Monocytes/macrophages

^99m^Tc-labeled nanobodies directed against the macrophage mannose receptor have been utilized for SPECT in a mouse model of rheumatoid arthritis [210]. Similarly, the macrophage folate receptor has been targeted with a ^68^Ga labeled PET probe to detect foreign body reactions in a mouse model [211]. Lastly, radiolabeled CCL2/MCP-1 (a chemoattractant for monocytes) was used to visualize sterile inflammation in rats [212], and atherosclerosis in rabbits [213].

#### CD40 on antigen-presenting cells (APC)

CD40 is required for activation of APCs, and binds to CD40L on Th lymphocytes. In the transient MCAO stroke model in mice, a monoclonal antibody against CD40 was conjugated to Cy5.5 and used to evaluate CD40 expression in the brain. No difference was seen in sham or stroke mice injected with Cy5.5-labeled control antibody, and sham or CD40 knockout stroke mice receiving Cy5.5-CD40, proving specificity. Increased fluorescent signal in the affected hemisphere was only seen in vivo in stroke mice injected with Cy5.5-CD40. Areas of CD40 signal corresponded to ischemic areas on TTC staining, and on histology CD40 signal partially co-localized with microglia and partially with cells in the vasculature [214].

## Damage Caused by Inflammation

### Demyelination

Demyelination is one of the consequences of neuroinflammation, especially in MS. While MRI has been used to evaluate myelination, all available techniques suffer from low specificity towards myelin [215,216].

Recently, a Congo red derivative named BMB, which shows spontaneous fluorescence, was synthesized and shown to specifically bind to myelin tracts on histology. In addition, this probe crossed the intact BBB and was sensitive to demyelination in two different dysmyelinating mutant mice, in brain slices from MS patients, and after radiolabeling with ^11^C for PET visualized myelin tracts in the brain of baboons [217]. A similar compound with higher solubility, named case imaging compound (CIC) was developed by the same group and shown to penetrate the BBB and bind to myelinated areas in the brain. ^11^C-labeled CIC was successfully used in longitudinal PET imaging in rats injected with lysolethicin to induce focal demyelination followed by remyelination [218].

**Figure 9:**
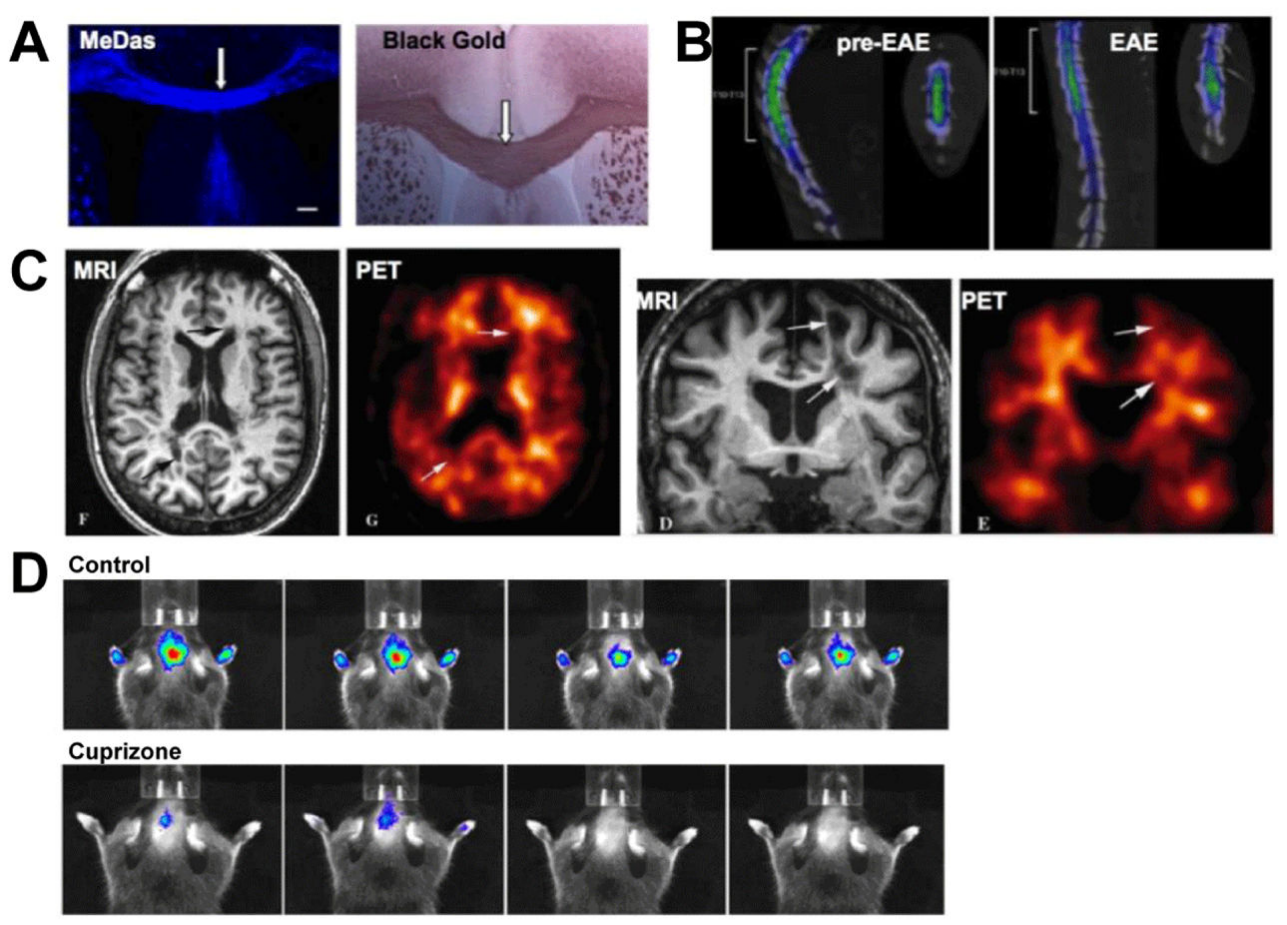
Molecular imaging of demyelination. (A) *In vitro* MeDas staining of myelin sheaths in the corpus callosum and comparison with Black Gold staining. (B) 3D PET and CT fusion image of a rat after injection of ^11^C-MeDas demonstrates decreased tracer uptake in the thoracic spinal cord in EAE versus preimmunized state. (C) PET-MR fusion imaging of demyelination with ^11^C-PIB in a patient with MS. MS plaques (arrows) and grey matter (arrowheads) sow decreased tracer uptake, suggesting reduced myelin content. (D) Optical near-infrared *in vivo* imaging of demyelination using the fluorescent compound DBT. In the cuprizone model of demyelination, reduced DBT uptake was detected compared to control mice. (Modified from Wu et al. [220,221], Stankoff et al. [227], and Wang et al. [223] with permission.)

Wu et al. [219] synthetized several PET agent candidates that somewhat resemble the chemical structure of luxol blue, a histological stain used to observe myelin. These compounds are thought to bind to myelin through direct and specific interaction with MBP. Their lead compound MeDAS was labeled with ^11^C, bound specifically to myelin (Figure 9A) and reliably distinguished PLP-Akt-DD mice, which develop hypermyelination, from wildtype mice [220]. In a study involving intracerebral injection of LPS to induce neuroinflammation without demyelination, focal demyelination, and EAE, ^11^C-MeDAS enhanced PET imaging demonstrated that agent uptake is unchanged after LPS injection, but uptake correlated with demyelination in lysophosphatidylcholine induced focal demyelination and EAE (Figure 9B) [221]. In a direct comparison study of ^11^C-CIC and ^11^C-MeDAS in the cuprizone mouse model, the latter showed superior correlation of de- and remyelination on PET [222].

For optical *in vivo* imaging on FRI, the compound DBT, a similar agent that emits fluorescence light in the near-infrared spectrum was capable of visualizing and quantifying myelination in hypermyelinated PLP-Akt-DD mice, hypomyelinated shiverer mice, and the process of demyelination and remyelination following treatment with cuprizone (Figure 9D) [223].

Case myelin compound (CMC) is another compound that specifically binds to myelin. Conjugated to DOTA-Gd, CMC bound to myelin in mouse brain sections but did not cross the BBB [224]. When DOTA-Gd-CMC was injected intraventricularly and MR imaging was performed at various time points after injection, MR signal corresponded to myelination on histology. In addition, DOTA-Gd-CMC enhanced MR imaging was capable of detecting focal demyelination induced by injection of lysophosphatidylcholine [225]. Similarly, a second MR probe, Gd-DODAS was also found to bind to myelin after intracerebral injection [226].

The thioflavine-T derivative Pittsburgh compound B (PIB) is well known to bind to beta-amyloid and has been used in clinical PET studies in Alzheimer’s patients. However, this compound was also shown to bind to myelin, identified demyelinated lesions on brain slices from MS patients, and was subsequently used for PET imaging of two patients with MS. There, lesions seen on MRI had lower ^11^C-PIB uptake compared to normal-appearing white matter, and DTPA-Gd enhancing lesions showed more ^11^C-PIB uptake than non-enhancing lesions, consistent with active inflammation and beginning demyelination versus chronic inflammation with more severe demyelination (Figure 9C) [227].

### Neuronal death

Probably the most important consequence of neuroinflammation is damage to neurons. In MS, neuronal loss correlates significantly with disease progression and irreversible disability [228,229]. In stroke, the “time is brain” paradigm underlines the importance of early treatment to prevent neuronal death and its clinical consequences [230].

**Figure 10:**
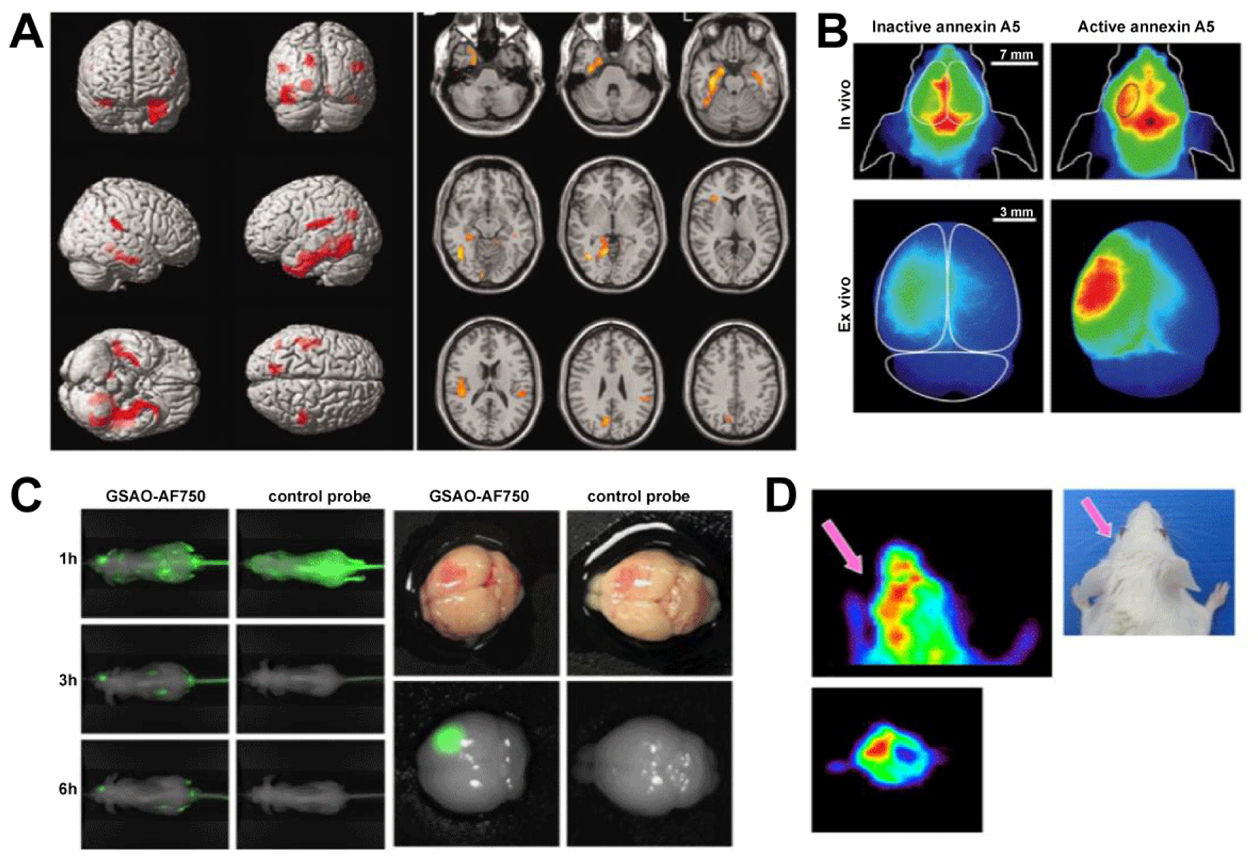
Molecular imaging of neuronal death. (A) PET-MR fusion imaging of a patient with Alzheimer’s disease using ^11^C-flumazenil. Regions with decreased tracer binding are shown in red on brain surface images, and yellow-red on axial images, and correspond to regions with greatest degree of neuronal loss in neuropathological studies. (B) *In vivo* and *ex vivo* near-infrared fluorescence annexin A5 imaging of ischemic stroke mice. Strong fluorescence is seen over the ischemic hemisphere only after injection of active Cy5.5-annexin A5. (C) *In vivo* and *ex vivo* imaging of mice with TBI using GSAO-AF750 or a nonspecific control probe. Specific uptake is seen in the damaged brain region. (D) PET using ^18^F-ML-10 to detect cell death in stroke mice *in vivo* demonstrates tracer uptake in the ischemic hemisphere. (Modified from Pascual et al. [233], Bahmani et al. [242], Xie et al. [250] and Reshef et al. [248] with permission.)

The central benzodiazepine receptor, which can be targeted with ^11^C-flumazenil enhanced PET, is expressed on neurons and has been shown to be good marker for neuron integrity in stroke [231,232]. In patients with Alzheimer’s disease, reduction of 11C-flumazenil binding corresponded precisely with areas of neuronal loss on postmortem histology (Figure 10A) [233]. In epilepsy, it revealed neuronal loss in the epileptogenic focus [234]. For MS, an ongoing trial is going to evaluate the prognostic value of neuronal damage as assessed by ^11^C-flumazenil PET in early MS. However, this technique might not be sensitive enough to detect neuronal loss in hyperacute ischemia [235], and could be confounded in diseases affecting the GABA neurotransmitter system such as epilepsy [236].

A more specific approach to imaging of neuronal death is targeting annexin-V. Annexin-V is a protein that binds with high affinity to apoptotic cells expressing phosphatidylserine (PS) on their cell surface [237]. Using ^99m^Tc labeled annexin-V, apoptosis was successfully detected on SPECT imaging in mouse models of fulminant hepatic apoptosis, acute cardiac transplant rejection, and cyclophosphamide treatment of a murine B cell lymphoma [238]. In a first clinical trial, ^99m^Tc-annexin-V showed increase uptake in 5 out of 18 patients after cardiac transplant, of whom all showed at least moderate transplant rejection and apoptosis on histology [239]. Since then, this probe has been used to detect neuronal damage corresponding to CT images in 12 acute ischemic stroke patients [240], and has demonstrated increased probe uptake in the cortex but not cerebellum of patients with Alzheimer’s disease [241]. For fluorescence imaging, Cy5.5 labeled annexin-V has been used in a mouse model of ischemic stroke to visualize neuronal death on FRI (Figure 10B) [242]. In addition, CLIO-labeled annexin-V for MR imaging [243], and ^18^F-labeled annexin-V for PET imaging [244] have been reported.

Caspases are cysteine-aspartic proteases that are crucially involved in cell death. They are grouped into an execution group (caspases 3, 6 and 7) and an initiation group (capases 8 and 10). Activation of the latter group is mediated via an intrinsic (release of cytochrome c from mitochondria) or extrinsic (via TNF, TRAIL, or FasL) pathway. Utilizing a far-red fluorescent pan-caspase inhibitor (NIR-VAD-fmk), Lawson et al. [245] identified activated (cleaved) caspases in brains of mice infected with prions using FRI, and this correlated with elevated levels of activated caspase-3 in the brain.

Other compounds belonging to the small molecule Aposense family have been designed. The small molecule DDC was radiolabeled with ^3^H for *ex vivo* autoradiography and detected neuronal death in rodent models of ischemic stroke and TBI [246,247]. Another small molecule, ML-10 was radiolabeled with ^18^F for PET imaging and studied in the MCAO mouse model of ischemic stroke. ^18^F-ML-10 was taken up into apoptotic but not necrotic cells *in vitro*, penetrated an intact BBB, increased signal in the affected hemisphere (Figure 10D), with areas with probe uptake correlated well with TUNEL^+^ areas on histology [248]. ^18^F-DFNSH enhanced PET was used in postnatal day 7 rats treated with ketamine anesthesia, which has been shown induce neuronal apoptosis [249]. Lastly, the compound GSAO rapidly accumulates in the cytoplasm of dying cells, coinciding with loss of plasma membrane integrity. Labeled with the near-infrared fluorochrome AF750, GSAO allowed for visualization of neuronal damage with FRI in a mouse model of TBI (Figure 10C), and signal correlated with apoptotic neurons on TUNEL stain on histology [250].

## Conclusion

Neuroinflammation is a highly complex process involving many different cell types and signaling molecules with the aim to control, regenerate, and repair. Under pathological conditions, however, neuronal loss and demyelination result in clinical disease and disability. The limited regenerative capacity of the brain makes early diagnosis and treatment the ultimate goal, and non-invasive molecular imaging of key players in the inflammation cascade holds promise for identification and quantification of the disease process before it is too late for effective therapeutic intervention. This would lead to early diagnosis and localization of pathology, better prediction of responders to treatment, and improved monitoring of treatment response. Additionally, many molecular imaging techniques are highly suitable to aid drug development in preclinical and clinical studies by allowing noninvasive longitudinal quantification of disease activity in the same subject.

Techniques such as TSPO PET imaging for microglia activation/monocyte infiltration, iron oxide particle enhanced MR imaging for phagocyte labeling, genetically engineered T-lymphocytes for PET, as well as myelin and neuronal death PET imaging have been already translated and tested in humans. In addition, preclinical methods such as MPO, MMP, and adhesion molecule imaging have already improved our understanding of the pathophysiology of many neuroinflammatory diseases. In the future, distinguishing neurotoxic from neuroprotective leukocyte populations, identification and visualization of key molecules and their receptors *in vivo*, and imaging the targets of future therapeutics together with key outcome predictors such as neuronal integrity to ensure success of a therapeutic intervention non-invasively will likely be the next goals of molecular imaging research. As we continue to make progress in utilizing molecular imaging technology to study and understand neuroinflammation, increasing efforts and investment should be made to bring more of these novel imaging agents from the “bench to bedside.”
